# The Effectiveness of Patient-Centred Medical Home-Based Models of Care versus Standard Primary Care in Chronic Disease Management: A Systematic Review and Meta-Analysis of Randomised and Non-Randomised Controlled Trials

**DOI:** 10.3390/ijerph17186886

**Published:** 2020-09-21

**Authors:** James Rufus John, Hir Jani, Kath Peters, Kingsley Agho, W. Kathy Tannous

**Affiliations:** 1 Translational Health Research Institute, Western Sydney University, Sydney, NSW 2560, Australia; H.Jani@westernsydney.edu.au (H.J.); K.Agho@westernsydney.edu.au (K.A.); k.tannous@westernsydney.edu.au (W.K.T.); 2 Rozetta Institute, Level 4, 55 Harrington Street, Sydney, NSW 2000, Australia; 3 School of Nursing and Midwifery, Western Sydney University, Sydney, NSW 2560, Australia; K.Peters@westernsydney.edu.au; 4 School of Science and Health, Western Sydney University, Sydney, NSW 2560, Australia; 5 School of Business, Western Sydney University, Sydney, NSW 2150, Australia

**Keywords:** patient-centred medical home, enhanced primary care, chronic disease management, collaborative care, meta-analysis

## Abstract

Patient-centred care by a coordinated primary care team may be more effective than standard care in chronic disease management. We synthesised evidence to determine whether patient-centred medical home (PCMH)-based care models are more effective than standard general practitioner (GP) care in improving biomedical, hospital, and economic outcomes. MEDLINE, CINAHL, Embase, Cochrane Library, and Scopus were searched to identify randomised (RCTs) and non-randomised controlled trials that evaluated two or more principles of PCMH among primary care patients with chronic diseases. Study selection, data extraction, quality assessment using Joanna Briggs Institute (JBI) appraisal tools, and grading of evidence using Grading of Recommendations, Assessment, Development, and Evaluation (GRADE) approach were conducted independently. A quantitative synthesis, where possible, was pooled using random effects models and the effect size estimates of standardised mean differences (SMDs) and odds ratios (ORs) with 95% confidence intervals were reported. Of the 13,820 citations, we identified 78 eligible RCTs and 7 quasi trials which included 60,617 patients. The findings suggested that PCMH-based care was associated with significant improvements in depression episodes (SMD −0.24; 95% CI −0.35, −0.14; I^2^ = 76%) and increased odds of remission (OR 1.79; 95% CI 1.46, 2.21; I^2^ = 0%). There were significant improvements in the health-related quality of life (SMD 0.10; 95% CI 0.04, 0.15; I^2^ = 51%), self-management outcomes (SMD 0.24; 95% CI 0.03, 0.44; I^2^ = 83%), and hospital admissions (OR 0.83; 95% CI 0.70, 0.98; I^2^ = 0%). In terms of biomedical outcomes, with exception to total cholesterol, PCMH-based care led to significant improvements in blood pressure, glycated haemoglobin, and low-density lipoprotein cholesterol outcomes. The incremental cost of PCMH care was identified to be small and significantly higher than standard care (SMD 0.17; 95% CI 0.08, 0.26; I^2^ = 82%). The quality of individual studies ranged from “fair” to “good” by meeting at least 60% of items on the quality appraisal checklist. Additionally, moderate to high heterogeneity across studies in outcomes resulted in downgrading the included studies as moderate or low grade of evidence. PCMH-based care has been found to be superior to standard GP care in chronic disease management. Results of the review have important implications that may inform patient, practice, and policy-level changes.

## 1. Introduction

Chronic diseases have contributed to increased mortality and morbidity worldwide with the disease burden accelerating across both developed and developing nations [1,2]. The Global Burden of Diseases (GBD) Study in 2017 reported that chronic diseases accounted for 41% of increased disability and 73% of all deaths [1,2]. Moreover, with increasing life expectancy and ageing population, the global prevalence of multiple chronic conditions or multimorbidity is also on the rise, further exacerbating complications in quality and delivery of care [3,4]. As a result, patients with one or more chronic diseases often experience poor mental and physical functioning with increased psychological distress affecting their overall health-related quality of life (HRQoL) [5,6]. In addition to negative health outcomes, chronic diseases also contribute to significant economic ramifications to both patients and health care system in the form of increased health care utilisation and costs of care [7,8].

The long-term nature of chronic diseases and complexities of care require health care systems, worldwide, to revisit guidelines on effective chronic disease management [7]. The health and economic repercussions of chronic diseases are partly connected to the fragmented design and delivery of health care systems to focus on “single disease framework” as opposed to a “whole-person approach” [9]. However, there has been an increasing advocacy towards shift from a reactive health care system to one that is proactive, enabling an integrated systems approach towards chronic disease management [10]. In view of this, the World Health Organisation (WHO) and other leading organisations have acknowledged the importance of primary care as an ideal setting to facilitate patient-centred care, which could result in better patient outcomes [11,12]. There is a large body of evidence suggesting that coordinated team-based approaches in primary care are effective in chronic disease management [13,14].

The patient-centred medical home (PCMH) model is one of the chronic care models (CCM) that has reportedly shown to provide a multidimensional solution to effectively managing chronic illness and multimorbidity in primary care [15]. This enhanced primary care model typically consists of a general practitioner (GP)-led care, as part of a multidisciplinary team (MDT) that aims to provide patient-centred care that is also comprehensive and coordinated, with emphasis on self-management and patient education [12]. There is a growing body of literature, particularly in United States and several parts of United Kingdom and other European countries, reporting the effectiveness of PCMH care models in improving biomedical [16,17], HRQoL [18,19], hospital [20,21], and economic outcomes [22] compared to standard GP care.

A comprehensive systematic review and meta-analysis of PCMH care published in 2013 [23] reported improvements in patient experiences and some reduction in health utilisation among patients with multimorbidity. However, the effect of PCMH models on patients with single-disease care management was not reviewed. Whilst the review focuses on clinical quality and processes of care, there was insufficient evidence to estimate biomedical outcomes and quality of life. In addition, the review also included patients from non-primary care settings such as tertiary care hospitals, thereby limiting understanding of the true effectiveness of PCMH model in primary care settings. The current review was warranted as there has been increased advocacy for PCMH-based care models resulting in a number of new studies evaluating PCMH models being published since 2013 [18,19,20,21].

A systematic review and meta-analysis was conducted to assess the effectiveness of PCMH-based models of care when compared to standard GP care in improving biomedical, hospital, and economic outcomes of primary care patients with one or more chronic diseases. The findings of this review may help inform guidelines and practices.

## 2. Methods

This review conformed to the Preferred Reporting Items for Systematic Reviews and Meta-Analyses (PRISMA) guidelines [24]. The systematic review protocol (CRD42018085378), registered in the International Prospective Register of Systematic Reviews (PROSPERO) database, has been published elsewhere [25].

### 2.1. Search Strategy

We conducted literature searches on electronic databases including MEDLINE, CINAHL, Embase, Cochrane library, and Scopus from inception until 31 March 2020. The search strategy and syntaxes were developed in collaboration with an experienced university librarian. The syntax explored a broad range of terms used in definitions of PCMH, collaborative care, chronic care models, RCTs, and Quasi trials (full electronic search strings are listed in Table A1). We supplemented electronic searches by hand-searching bibliographies of several key systematic reviews [23,26,27,28] and retrieved studies to identify any relevant articles missed by the search strategy. Endnote (Version X9, Thompson Reuters, New York, NY, USA) software was used for reference management.

### 2.2. Eligibility Criteria and Study Selection

**Figure 1:**
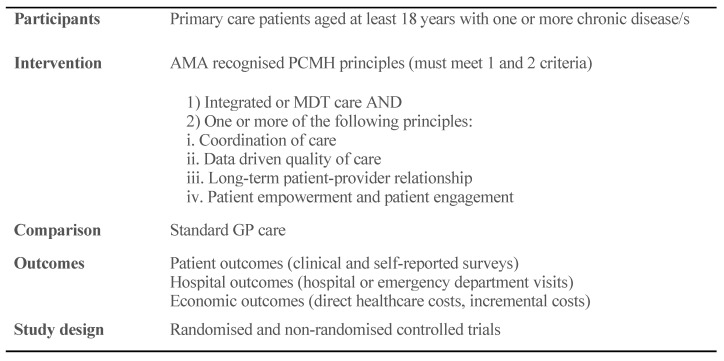
Summary of Population, Interventions, Comparators, Outcomes, and Study designs (PICOS) components. Outcomes included but not limited to patient, hospital, and economic outcomes.

A detailed inclusion and exclusion criteria along with explanation of core PCMH principles is reported elsewhere [25]. A summary of Population, Interventions, Comparators, Outcomes, and Study designs (PICOS) framework is presented in Figure 1. Two reviewers (JRJ and KP) independently screened the titles and abstracts of all articles for eligibility. Following the title and abstract screening, a full text screening was conducted on articles which passed the title and abstract screening by two reviewers (JRJ and HJ) independently. Discrepancies were resolved and clarified through discussion.

### 2.3. Data Extraction

Data extraction of included articles was carried out independently by two reviewers (JRJ and HJ) using Excel spreadsheet (Microsoft Excel, Microsoft Corporation). Data extracted from included articles included key characteristics: first author and publication year; country of origin; sample size, age, and gender distribution; chronic disease profile; baseline characteristics reported as mean (SD) or proportions; PCMH components implemented; duration of follow-up; and outcomes. Whilst data extraction was performed using a customised spreadsheet, the Centre for Reviews and Dissemination’s (CRD) guidance for undertaking reviews in health care was followed [29]. Authors of studies with missing data were contacted by email up to two times; however, no response was received.

### 2.4. Quality Assessment and Risk of Bias

Two reviewers (JRJ and HJ) independently evaluated the methodological validity of included articles using relevant Joanna Briggs Institute (JBI) critical appraisal checklists (RCTs, quasi trials, and economic evaluations) [30,31]. Quality of studies were rated as good (≥8), fair (6–7), or poor (≤5) based on the summary scores. We also used risk of bias in non-randomised studies of interventions (ROBINS-I) tool to supplement JBI appraisal for non-randomised trials [32]. Additionally, the quality of evidence across included studies reporting similar outcomes was determined by applying the Grading of Recommendations Assessment, Development and Evaluation (GRADE) criteria [33]. The overall GRADE quality of evidence from the tables takes into account three factors which include (i) the average quality across the studies for each particular outcome, (ii) the level of heterogeneity between the studies, and (iii) the total number of studies reporting a particular outcome.

### 2.5. Outcomes

Outcomes identified from the studies include changes in mean differences or proportion of patients achieving recommended levels in

(1). Biomedical outcomes—blood pressure (BP); glycated haemoglobin (HbA1c); low density lipoprotein cholesterol (LDL-C); high density lipoprotein cholesterol (HDL-C); and serum total cholesterol.
(2). Self-reported health assessments (using validated questionnaires)—depression; HRQoL (overall, mental and physical functioning components); and self-management.
(3). Health utilisation outcomes—hospital admissions; emergency department visits; and medications use.
(4). Economic outcomes—incremental cost-effectiveness ratio (ICER) which is defined as the difference in total cost of an intervention (compared to standard care) divided by the difference in health outcome measure [22].

### 2.6. Data Analysis

Data of included studies were pooled together using the inverse-variance method of random-effects meta-analysis [34]. Standardised mean differences (SMD) for continuous data and odds ratios (ORs) for dichotomous data, with 95% confidence intervals (CI), were calculated and graphically presented as forest plots. Statistical heterogeneity was calculated using I^2^ and Cochran’s Q statistics. Subgroup analyses were considered for outcomes with substantial heterogeneity (I^2^ ≥ 85%). Publication bias for outcomes with at least 6 studies was assessed using funnel plots and Egger’s test of asymmetry [35]. All analyses were conducted using RevMan version 5.3 (The Nordic Cochrane Centre, Copenhagen, Denmark) and R version 4.0 software (R Foundation for Statistical Computing, Vienna, Austria).

## 3. Results

### 3.1. Literature Search

**Figure 2:**
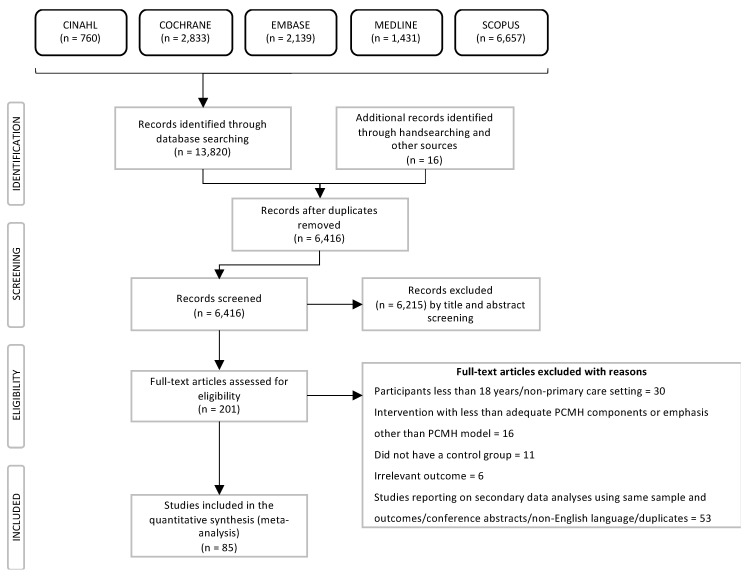
Preferred Reporting Items for Systematic Reviews and Meta-Analyses (PRISMA) Flowchart.

The electronic database search resulted in 13,820 citations and an additional 16 citations from hand searching key systematic reviews. After exclusion of duplicate records, 6416 articles were screened by titles and abstracts with 201 articles determined to be eligible for full-text assessment. Of these, 85 studies met the eligibility criteria and were included in our systematic review. Flowchart of the selection process from initial identification to inclusion is shown in Figure 2. Main reasons for exclusion included patients treated in non-primary care settings, not meeting minimum PCMH components or focused on intervention other than PCMH model, lack of control group, and other reasons (list of excluded articles; see Table A2).

### 3.2. Descriptive Data Synthesis

The characteristics of included studies are presented in the Appendix A Table A3 and Table A4. Of the 85 studies included in the review, 78 studies were RCTs [13,14,16,18,19,20,22,36,37,38,39,40,41,42,43,44,45,46,47,48,49,50,51,52,53,54,55,56,57,58,59,60,61,62,63,64,65,66,67,68,69,70,71,72,73,74,75,76,77,78,79,80,81,82,83,84,85,86,87,88,89,90,91,92,93,94,95,96,97,98,99,100,101,102,103,104,105,106] and 7 studies were of non-RCTs, including quasi trials [17,21,107,108] or cohort studies with a control group [109,110,111]. The 85 studies enrolled a total of 60,617 patients with sample sizes ranging from 40 to 8366. Whilst 79 studies had sufficient data for quantitative data synthesis, 6 studies [81,85,95,97,103,107] did not have usable data and therefore, the findings were narratively summarised.

The common inclusion criteria for all 85 studies was primary care patients with diagnosis of one or more chronic conditions, whereas the predominant reason for exclusion was patients with cognitive impairment and terminal illness. In terms of the chronic disease profile of the participants in the included articles, 46% of articles were based on participants with single chronic condition whereas 54% reported on one or more conditions. The most prevalent conditions were mental illness (59%), type 2 diabetes (33%), cardiovascular diseases (CVD) including hypertension (20%), musculoskeletal disorders (6%), and chronic obstructive pulmonary disease (COPD) (6%) (Table A3 and Table A4).

More than half the studies (52%) were conducted in the United States. The mean age of patients ranged between 30 and 83 years. In terms of gender distribution, most of the studies had slightly more women than men, except for studies conducted in Veterans Affairs (VA) primary care settings [16,50,52,53,56]. The duration of follow-up varied from 3 to 48 months. Out of 85 articles included for review, in addition to MDT care, 95% of studies reported coordinated care, patient engagement and education, and self-management; 20% reported continuity of care and long-term patient provider relationship; and only 9% of studies included data driven quality of care (Table A3 and Table A4).

### 3.3. Quality Assessment and Risk of Bias

Quality assessment and risk of bias for individual studies are reported in the Appendix A Table A5, Table A6, Table A7, Table A8. The overall quality of studies ranged from “fair” to “good” by meeting at least 60% of items on the checklist. Two studies [62,104] were rated as poor due to general lack of information on randomisation, unclear methodology, and clarity of results. Given the nature of PCMH-based intervention, most trials employed a cluster randomisation method where a group of patients were seen by the same GP or same general practice providing PCMH care. Thereby, blinding of patients or GPs was not applicable and, as a result, items related to blinding were not necessarily graded down. However, only 32 studies reported blinding of outcome assessment whilst other studies were graded down in quality. The quality of evidence across included studies assessed using GRADE approach is presented in Table 1. 

### 3.4. Depression Outcomes

**Figure 3:**
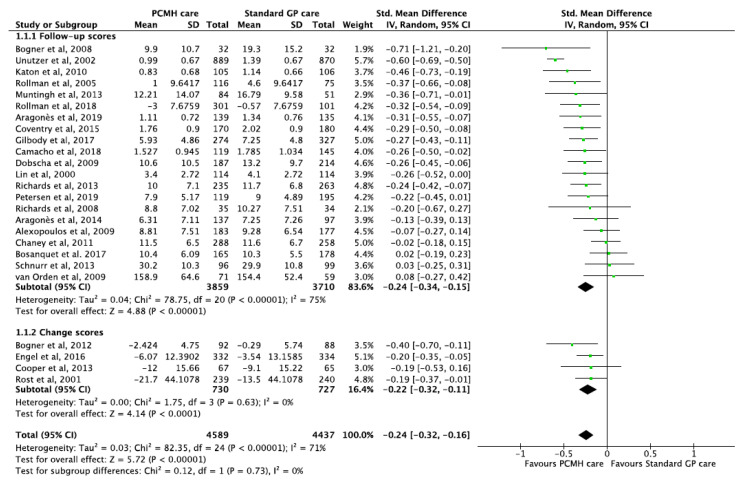
Forest plots of depression outcomes between the PCMH care and Standard GP care.

Meta-analysis of thirty-one studies [13,14,18,19,36,38,40,42,43,46,50,51,53,55,57,63,67,68,70,76,78,83,84,86,87,88,91,93,100,102,109] of patients with minor or major depression episodes after PCMH-based care reported significant improvement in depression scores compared to patients with standard primary care. With the exceptions of three studies [46,91,102], twenty-two studies reporting changes in mean differences (continuous data) of depression scores showed significant reduction with a pooled SMD of −0.24 (95% CI −0.35, −0.14; *p*-value < 0.001) (Figure 3).

Six studies reported that PCMH care was associated with significantly increased odds of remission of depression with pooled OR 1.79 (95% CI 1.46, 2.21; *p*-value < 0.001) (Figure 3). Additionally, one other study [85] reported significant improvements among patients with anxiety and mood disorders with an effect size of 0.30 (95% CI 0.05, 0.55; *p*-value = 0.02) compared to standard care. Given most studies consistently reported improvements, the GRADE of evidence was classified as moderate quality (Table 1).

### 3.5. Quality of Life Outcomes

**Figure 4:**
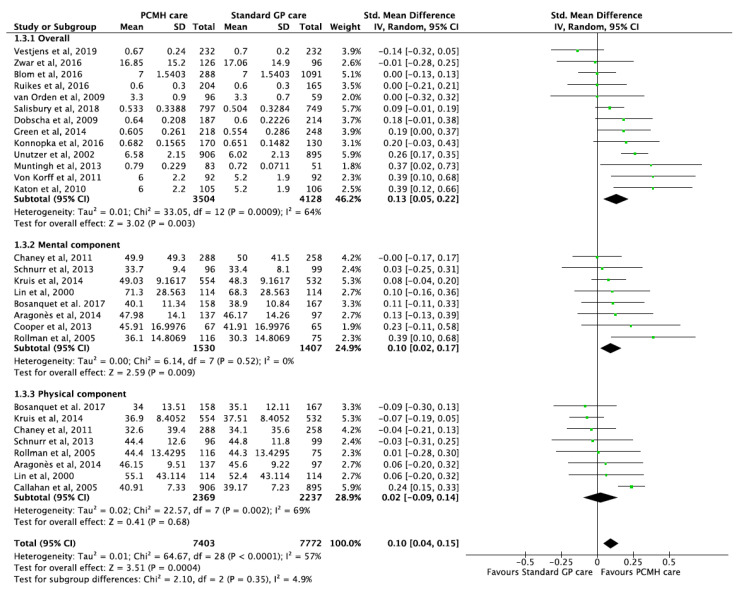
Forest plots of Quality of life (QoL) outcomes between the PCMH care and Standard GP care.

Twenty-two studies [18,19,21,22,41,46,49,50,51,53,59,68,72,76,86,89,91,100,102,105,106,108] evaluated the effectiveness of PCMH-based care on HRQoL (overall, physical component and mental component). Patients enrolled in PMCH-based care reported small but significant improvements in HRQoL compared to standard care with a pooled SMD of 0.10 (95% CI 0.04, 0.15; *p*-value < 0.001) (Figure 4). Additionally, one other study [85] reported significant improvements with an effect size of 0.38 (95 % CI 0.13, 0.63; *p*-value = 0.003). Moderate heterogeneity was observed among included studies (I^2^ = 57%), but test for sub-group differences were not significant. The GRADE of evidence was classified as moderate quality (Table 1).

### 3.6. Blood Pressure Outcomes

**Figure 5:**
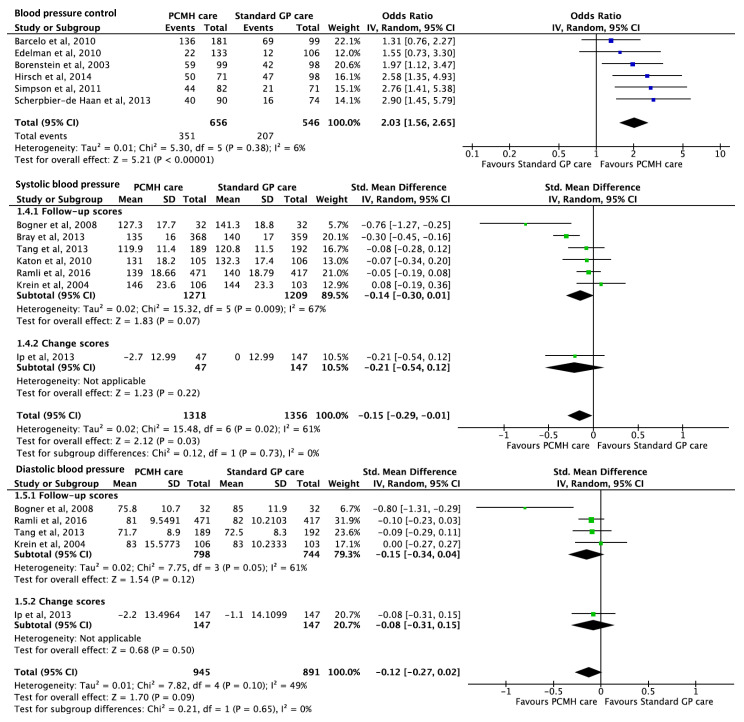
Forest plots of blood pressure outcomes between the PCMH care and Standard GP care. BP control refers to blood pressure levels within the guideline’s recommended range.

Thirteen studies [16,17,39,42,45,61,64,68,71,82,90,94,96] reported on the effect of PCMH care on blood pressure outcomes. Six studies reported that PCMH care was associated with significantly increased odds of BP control with pooled OR 2.03 (95% CI 1.56, 2.65; *p*-value < 0.001) (Figure 5). Seven studies reported significant improvements in systolic blood pressure (SBP), in favour of PCMH care, with pooled estimates of SMD −0.15 (95% CI −0.29, −0.01; *p*-value = 0.03). Similar reduction was observed across five studies reporting on diastolic blood pressure (DBP), but the pooled estimate of SMD −0.12 (95% CI −0.27, 0.02; *p*-value = 0.09) failed to meet significance (Figure 5). The GRADE of evidence was classified as moderate quality (Table 1).

### 3.7. Glycated Haemoglobin Outcomes

**Figure 6:**
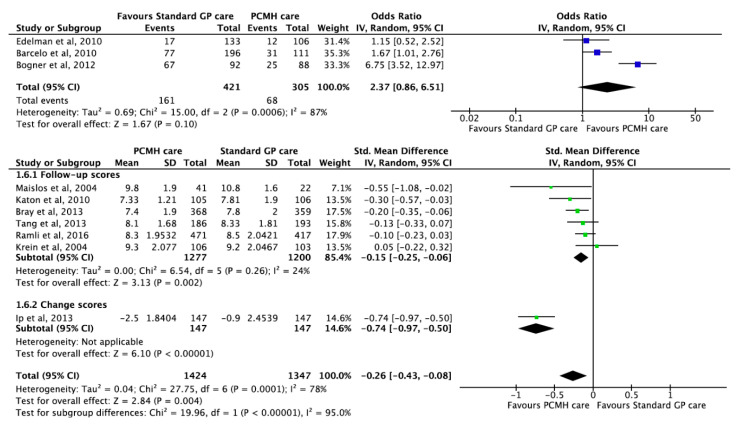
Forest plots of HbA1c outcomes between the PCMH care and Standard GP care. HbA1c control refers to HbA1c levels within the guideline’s recommended range.

Ten studies [16,17,39,43,64,68,71,77,82,96] reported on the effect of PCMH care on HbA1c outcomes. HbA1c levels were recorded among patients with a positive diagnosis of Type 2 diabetes. Three studies reported that PCMH care was associated with increased odds of glycaemic control with pooled OR 2.37 (95% CI 0.86, 6.51; *p*-value = 0.100). However, the pooled estimate was not statistically significant (Figure 6). The substantial heterogeneity of 87% in the three studies reporting ORs was due to a shorter follow-up duration of three months reported by Bogner et al. [43] compared to the other two studies which had follow-up duration of 12 to 13 months. Seven studies reported significant improvements in HbA1c, in favour of PCMH care with pooled estimates of SMD −0.26 (95% CI −0.43, −0.08; *p*-value = 0.004) (Figure 6). Given the substantial amount of heterogeneity, the GRADE of evidence was classified as low quality (Table 1).

### 3.8. Cholesterol Outcomes

**Figure 7:**
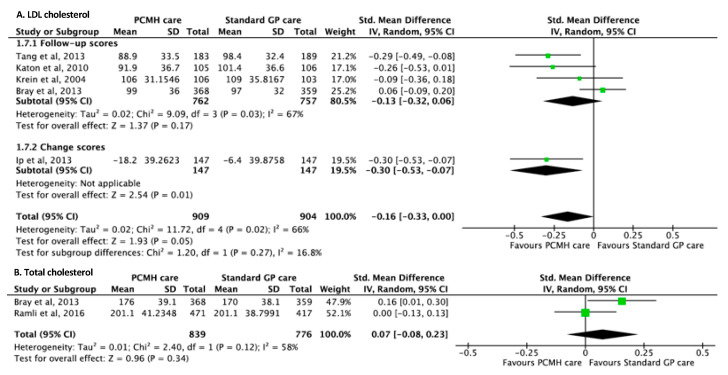
Forest plots of (**A**) LDLcholesterol and (**B**) Total cholesterol outcomes between the PCMH care and Standard GP care.

For LDL-cholesterol outcomes, five studies [17,64,68,71,96] reported significant improvements in favour of PCMH care with pooled SMD of −0.16 (95% CI −0.33, −0.00; *p*-value = 0.05) compared to standard GP care. Test for subgroup difference between follow-up and change scores showed no statistical significance (I^2^ = 16.8%, *p*-value = 0.27) (Figure 7A). For total cholesterol outcomes, two studies [17,82] reported a non-significant increase in total cholesterol with a pooled SMD of 0.07 (95% CI −0.08, 0.23; *p*-value = 0.34) (Figure 7B). The GRADE of evidence of both LDL and total cholesterol outcomes were classified as low quality given the limited number of studies (Table 1).

### 3.9. Hospital Admissions

**Figure 8:**
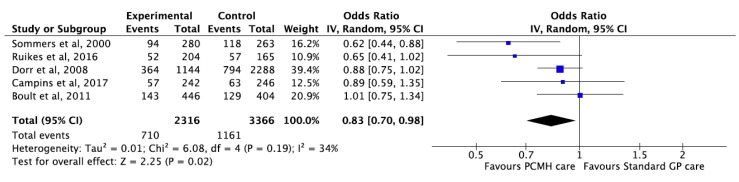
Forest plot for hospital admissions between PMCH care and Standard GP care.

Five studies [20,21,48,54,111] reported that PCMH care was associated with significant reduction in hospital admissions compared to standard care with pooled OR 0.83 (95% CI 0.70, 0.98; *p*-value = 0.02) (Figure 8). Additionally, one study [110] reported a reduction in mean hospital admission rates related to diabetic complications 12 months after PCMH based care compared to standard care. Nonetheless, the change in mean difference failed to meet statistical significance. The GRADE of evidence was classified as moderate quality (Table 1).

### 3.10. Self-Management Outcomes

**Figure 9:**
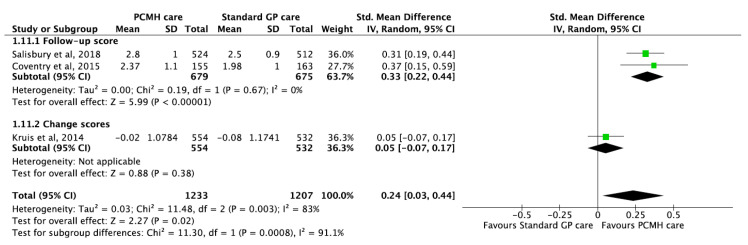
Forest plots of self-management outcomes (Patient Assessment of Care for Chronic Conditions (PACIC) scores) between the PCMH care and Standard GP care.

Three studies [14,72,89] reported significant improvements in self-management scores in favour of PCMH care compared to standard care with pooled estimates of SMD 0.24 (95% CI 0.03, 0.44; *p*-value < 0.001) (Figure 9). Given the substantial amount of heterogeneity (I^2^ = 83%), the GRADE of evidence was classified as low quality (Table 1).

### 3.11. Economic Outcomes

**Figure 10:**
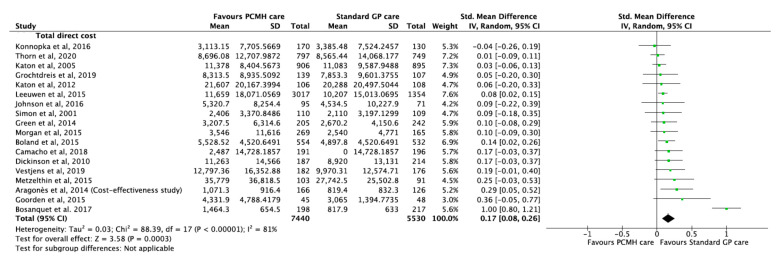
Forest plots of incremental cost of intervention between the PCMH care and Standard GP care.

A total of 18 studies [13,22,37,44,46,52,58,59,60,65,66,69,73,79,80,92,98,108] reported cost-effectiveness of PCMH-based models of care compared to standard care. To avoid bias in analysis, all currencies were converted to US Dollars at the time of the respective trials and cost effectiveness was measured in terms of incremental cost of intervention. The incremental cost of PCMH care was small but significantly higher than standard care with a pooled estimate of 0.17 (95% CI 0.08, 0.26; *p*-value < 0.001) (Figure 10). The substantial heterogeneity of 81% was due to higher costs of intervention reported by Bosanquet et al. [46]. The GRADE of evidence was classified as low quality (Table 1).

A summary of results from meta-analyses (where possible) and individual studies from randomised and non-randomised controlled trials are presented in Table 2.

### 3.12. Publication Bias

**Figure 11:**
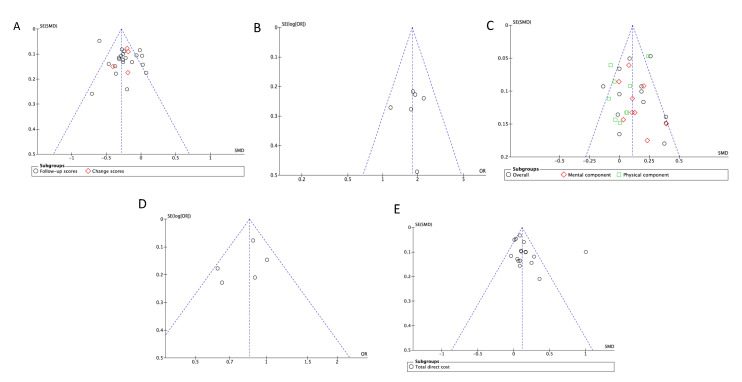
Funnel plots assessing asymmetry of depression, QoL, hospital admissions, and cost outcomes between the PCMH care and Standard GP care. (**A**)—Depression (SMD); (**B**)—Depression (OR); (**C**)—Quality of Life (SMD); (**D**)—Hospital admissions (OR); (**E**)—Direct costs.

**Figure 12:**
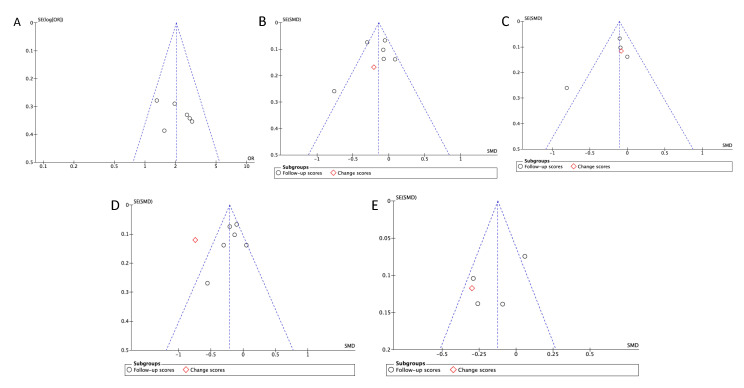
Funnel plots assessing asymmetry of biomedical outcomes between the PCMH care and Standard GP care. (**A**)—Blood pressure (SMD); (**B**)—Systolic blood pressure (OR); (**C**)—Diastolic blood pressure (SMD); (**D**)—HbA1C (OR); (**E**)—LDL cholesterol.

Six or more articles with similar outcomes were inspected for publication bias visually by using funnel plots and statistically by determining the significance from Egger’s test of asymmetry. Visual inspection of included studies reporting similar outcomes did not indicate any obvious sign of asymmetry (Figure 11 and Figure 12). Consistent with visual findings, no evidence of publication bias was detected with Egger’s test, as all outcomes had *p* > 0.05, showing evidence of funnel plot symmetry (Table 2).

## 4. Discussion

### 4.1. Summary of Findings

This systematic review comprehensively summarised current evidence on the effectiveness of PCMH-based models on chronic disease management among primary care patients. Compared to standard GP care, PCMH-based care led to significant improvements in depression episodes, quality of life, HbA1c, LDL cholesterol, hospital admissions, and self-management outcomes. Whilst PCMH care was significantly associated with increased odds of blood pressure control, reductions in both pooled estimates of SBP and DBP were not statistically significant. In contrast, the findings suggest that PCMH-based interventions have higher costs and was not cost-effective when compared to standard care. Additionally, the narrative synthesis of studies also corroborated with pooled estimates of the meta-analyses.

### 4.2. Consistency with Other Systematic Reviews

The most commonly reported PCMH principles in the included studies were patient engagement through education and self-management, and care coordination in addition to team-based care. Findings of this review, underscoring these PMCH elements in primary care, are consistent with previous systematic reviews reporting quality of care and overall patient experiences [26,112]. In terms of study outcomes, depression and HRQoL were frequently reported outcomes in the included studies. Systematic reviews focusing on depression outcomes as a result of collaborative care reported similar improvements, which were consistent with our pooled estimates of SMDs and ORs [113,114]. Similarly, our review showed small but significant improvements in the self-reported HRQoL and self-management scores, which is consistent with previous reviews [115,116]. Variabilities in the duration of intervention and baseline severity of chronic illness may explain smaller pooled estimates of HRQoL outcome.

Changes in biomedical outcomes are common measures employed in evaluating the effectiveness of chronic disease management interventions. With the exception of total cholesterol outcomes, findings of our studies were consistent with previous reviews [117,118], showing improvements in biomedical outcomes in favour of PCMH-based care compared to standard care. In terms of cost-effectiveness of PCMH-based models, some meta-analytic reviews on economic evaluations showed that PCMH care was associated with decreases in total costs compared to standard care [119,120]. However, our review supports evidence from prior reviews [115,121], suggesting that PCMH-based care was not associated with improvement in cost outcomes compared to standard care. This discordance could be due to the variability in the initial and sustained amount of costs incurred as a result of additional staffing and other infrastructure as well as the sample of patients and their comorbidity profile in the included trials [121].

### 4.3. Strengths and Limitations

Quality assessment for risk of bias was assessed within and across studies of similar outcomes. As aforementioned, blinding of patients and GPs was not possible due to the nature of intervention and design of trials, as reported in other systematic reviews conducted in primary care settings [114,122]. A substantial amount of heterogeneity was observed for measures of depression, HbA1c, and incremental cost of intervention, justifying the choice of random effects model. Higher heterogeneity is expected when pooling results of complex interventions, given the varying levels of intensity of different interventions, follow-up times, chronic disease profile of participants, and country’s primary care setting [115]. Nonetheless, pooled estimates are to be interpreted with caution given unexplained variation observed in outcomes with higher heterogeneity. The review did not consider unpublished data or non-English language studies given the exhaustive number of citations identified. This may have had potential impact on effect size estimates.

Whilst previous reviews and meta-analyses on collaborative care for either single specific disease or multimorbidity have been studied, this review provides a comprehensive current evidence with quantitative synthesis on the effectiveness of PCMH-based care models exclusively on primary care patients with one or more chronic diseases. Other strengths include a registered and published protocol, with a peer-reviewed search strategy, conducted on a wide range of electronic databases.

### 4.4. Patient, Provider, and Policy-Level Implications and Future Directions

Findings of our systematic review have important implications at patient, practice, and policy-level. The evidence may inform patients on the enhanced biomedical outcomes and quality of life resulting from improved education and self-management support. The transformational changes at practice level may enable GPs to better target and deliver care according to the level and complexity of different patients [123]. Additionally, our study findings may also impact policy and implementation guidelines given the growing advocacy towards patient-centred care. Future research should focus on evaluating sustained benefits of PCMH-based care as well as supporting holistic experiences of patients receiving patient-centred care.

## 5. Conclusions

Current evidence suggests that PCMH-based care showed significant improvements in depression, HRQoL, self-management, biomedical, and health utilisation outcomes compared to standard GP care. Whilst studies included for pooled estimates showed consistent trend for several outcomes, high heterogeneity in some outcomes resulted in low to moderate grade of evidence, limiting firmer conclusion from the pooled evidence. Further research is needed to evaluate the long-term cost-effectiveness of PCMH-based care after the initial higher costs incurred for intervention, which may prove to be more cost-effective than standard care.

## Figures and Tables

**Table 1:** Grading of Recommendations, Assessment, Development, and Evaluation (GRADE) assessment of randomised controlled trials reporting effectiveness of patient-centred medical home (PCMH) vs. standard general practitioner (GP) care on outcomes of interest.

Outcomes	No of Studies	Risk of Bias	Inconsistency	Indirectness	Imprecision	Publication Bias	GRADE Quality of Evidence ^þ^
Depression	31	Serious	Serious	Not serious	Not serious	Undetected	Moderate ^‡^
Quality of Life	21	Serious	Not serious	Not serious	Not serious	Undetected	Moderate ^‡^
Blood pressure	13	Serious	Not serious	Not serious	Not serious	Undetected	Moderate ^‡^
Glycated Hemoglobin	9	Serious	Serious	Not serious	Not serious	Undetected	Low ^‡¶^
LDL Cholesterol	4	Serious	Serious	Not serious	Not serious	Undetected	Low ^‡¶^
HDL Cholesterol	1	Serious	-	Not serious	Not serious	Undetected	Low ^†‡^^
Total Cholesterol	2	Serious	-	Not serious	Not serious	Undetected	Low ^‡^^
Hospital admissions	5	Serious	Not serious	Not serious	Not serious	Undetected	Moderate ^‡^
Self-management (PACIC scores)	3	Serious	Serious	Not serious	Not serious	Undetected	Low ^‡¶^
Cost-effectiveness	19	Serious	Serious	Not serious	Not serious	Undetected	Low ^‡¶^

^þ^ High quality: Further research is very unlikely to change our confidence in the estimate of effect; Moderate quality: Further research is likely to have an important impact on our confidence in the estimate of effect and may change the estimate; Low quality: Further research is very likely to have an important impact on our confidence in the estimate of effect and is likely to change the estimate; Very low quality: We are very uncertain about the estimate; LDL—Low Density Lipoprotein; HDL—High Density Lipoprotein; PACIC—Patient Assessment of Care for Chronic Conditions; ^‡^ Most studies did not blind participants or personnel as it was not practical. Therefore, we did not downgrade for these risks/uncertainties. However, studies not reporting blinding of outcome assessment were downgraded in quality; ^¶^ Significant level of heterogeneity within results (I^2^ between 80–90%); ^^^ Single study—Inconsistency not applicable; ^†^ Because of the nature of the quasi-experimental designs risk of bias is unavoidable.

**Table 2:** Summary of findings from meta-analyses (where possible) or individual studies from randomised and non-randomised controlled trials.

Outcome	No of Studies	No of Participants	Effect Size (95% CI)	*p*-Value	Q Statistic	I^2^	Egger’s Test *p*-Value ^‡^	Citations	Figure
**Randomised controlled trials**									
Depression	24 6	7255 1520	SMD −0.24 (−0.35, −0.14) OR 1.79 (1.46, 2.21)	<0.001 <0.001	78.3 3.58	76% 0%	0.275 0.608	[13,14,18,19,36,38,40,42,43,46,50,51,53,55,57,63,67,68,70,76,78,83,84,86,87,88,91,93,100,102,109]	Figure 3
Quality of Life	22	12,370	SMD 0.12 (0.09, 0.15)	<0.001	57.38	51%	0.556	[18,19,21,22,41,46,49,50,51,53,59,68,72,76,86,89,91,100,102,105,106,108]	Figure 4
Blood pressure									
BP control	6	1202	OR 2.03 (1.56, 2.65)	<0.001	5.30	6%	0.347	[16,39,42,45,61,64,68,71,82,90,94,96]	Figure 5
Systolic BP	6	1947	SMD −0.08 (−0.17, 0.01)	0.09	8.97	44%	0.737		
Diastolic BP	5	1836	SMD −0.12 (−0.27, 0.02)	0.10	7.82	49%	0.260		
Glycated haemoglobin								[16,39,43,64,68,71,77,82,96]	Figure 6
Glycaemic control	3	726	OR 2.37 (0.86, 6.51)	0.001	15.00	87%	NA		
*HbA1c*	6	2044	SMD −0.21 (−0.30, −0.12)	<0.001	27.75	82%	0.405		
LDL Cholesterol	4	1086	SMD −0.25 (−0.37, −0.13)	<0.001	1.64	0%	NA	[64,68,71,96]	Figure 7A
Total Cholesterol	1	888	SMD 0.00 (−0.13, 0.13)	1.00	NA	NA	NA	[82]	Figure 7B
Hospital admissions	3	4770	OR 0.90 (0.80, 1.03)	0.12	0.67	0%	NA	[20,48,54]	Figure 8
Self-management (PACIC scores)	3	2440	SMD 0.24 (0.03, 0.44)	0.02	11.48	83%	NA	[14,72,89]	Figure 9
Cost-effectiveness	17	12,612	SMD 0.17 (0.07, 0.26)	0.001	87.84	82%	0.206	[13,22,37,44,46,52,58,59,60,65,66,69,73,79,80,92,98]	Figure 10
**Non-randomised trials**									
Depression	1	314	SMD −0.22 (−0.45, 0.01)	0.06	NA	NA	NA	[109]	Figure 3
Quality of Life	2	833	SMD −0.08 (−0.21, 0.06)	0.28	0.94	0%	NA	[22,108]	Figure 4
Blood pressure									Figure 5
Systolic BP	1	727	SMD −0.30 (−0.45, −0.16)	<0.001	NA	NA	NA	[17]	
Glycated haemoglobin	1	727	SMD −0.20 (−0.35, −0.06)	0.006	NA	NA	NA	[17]	Figure 6
LDL Cholesterol	1	727	SMD 0.06 (−0.09, 0.20)	0.43	NA	NA	NA	[17]	Figure 7
HDL Cholesterol	1	727	SMD 0.15 (0.00, 0.29)	0.05	NA	NA	NA	[17]	-
Total Cholesterol	1	727	SMD 0.16 (0.01, 0.30)	0.04	NA	NA	NA	[17]	Figure 8
Hospital admissions	2	912	OR 0.63 (0.48, 0.83)	0.001	0.02	0%	NA	[21,111]	Figure 9
Cost-effectiveness	1	358	SMD 0.19 (−0.01, 0.40)	0.07	NA	NA	NA	[108]	Figure 10

NA—not applicable; SMD—Standard Mean Difference; OR—Odds ratio; ^‡^ Egger’s test was conducted only for outcomes with at least 6 studies. Note: The slight discrepancy in the effect sizes in this table to that reported in the manuscript and figures is because the effects sizes are classified based on their study design. I^2^ describes the percentage of total variation across studies that is due to heterogeneity rather than chance. A value of 0% indicates no observed heterogeneity, and larger values show increasing heterogeneity.

## Appendix A

**Table A1:** Search strategy.

No	Search Terms
1	PCMH.tw.
2	(patient-centred adj medical adj home *).tw.
3	(patient adj centred adj medical adj home *).tw.
4	(patient-centered adj medical adj home *).tw.
5	(patient adj centered adj medical adj home *).tw.
6	(Medical adj home *).tw.
7	(Home adj based adj care).tw.
8	(home adj based adj model).tw.
9	(Health adj home *).tw.
10	(Health adj care adj home *).tw.
11	(Health-care adj home *).tw.
12	(Patient adj centred adj care).tw.
13	(Patient-centred adj care).tw.
14	(Patient adj centered adj care).tw.
15	(Patient-centered adj care).tw.
16	(Patient adj focused adj care).tw.
17	(Patient-focused adj care).tw.
18	(Integrated adj primary adj care).tw.
19	(Integrated adj care).tw.
20	(Integrated adj health adj care).tw.
21	(Integrated adj service *).tw.
22	(Integrated adj delivery).tw.
23	(Team-based adj care).tw.
24	(multidisciplinary adj care *).tw.
25	(care adj team).tw.
26	(care adj coordination).tw.
27	(coordinated adj care).tw.
28	(coordinated adj health adj care).tw.
29	(coordinated adj primary adj care).tw.
30	(collaborative adj practice).tw.
31	(Collaborative adj care).tw.
32	(Advanced adj primary adj care).tw.
33	(enhanced adj primary adj care).tw.
34	(augmented adj care).tw.
35	(augmented adj service *).tw.
36	(guided adj care).tw.
37	(chronic adj care adj model *).tw.
38	(Patient adj aligned adj care adj team).tw.
39	(patient adj care adj team).tw.
40	1 or 2 or 3 or 4 or 5 or 6 or 7 or 8 or 9 or 10 or 11 or 12 or 13 or 14 or 15 or 16 or 17 or 18 or 19 or 20 or 21or 22 or 23 or 24 or 25 or 26 or 27 or 28 or 29 or 30 or 31 or 32 or 33 or 34 or 35 or 36 or 37 or 38 or 39
41	(primary adj health adj care).tw.
42	(family adj practice *).tw.
43	(primary adj care *).tw.
44	(community adj network *).tw.
45	(health adj care adj coalitions).tw.
46	(chronic adj care *).tw.
47	(primary adj physician *).tw.
48	(primary adj care adj physician *).tw.
49	(general adj practice *).tw.
50	(general adj physician *).tw.
51	(general adj practitioner *).tw.
52	(community adj based adj provider *).tw.
53	(community adj practice).tw.
54	(community adj care).tw.
55	(preventive adj service *).tw.
56	(patient adj care).tw.
57	Adult *.tw.
58	(middle adj age *).tw.
59	geriatric.tw.
60	(geriatric adj practice).tw.
61	elder *.tw.
62	exp Chronic Disease/
63	(Chronic adj disease *).tw.
64	(Chronic adj illness *).tw.
65	exp COMORBIDITY/
66	comorbid *.tw.
67	multimorbid *.tw.
68	exp Diabetes Mellitus/
69	((Diabetes adj mellitus) or Diabet *).tw.
70	exp ASTHMA/
71	Asthma *.tw.
72	exp ARTHRITIS/
73	Arthritis.tw.
74	exp Back Pain/
75	(Back adj pain).tw.
76	exp Cardiovascular Diseases/
77	(cardiovascular adj disease *).tw.
78	(Heart adj disease *).tw.
79	exp Neoplasms/
80	cancer *.tw.
81	(malignant adj neoplasm *).tw.
82	exp Pulmonary Disease, Chronic Obstructive/
83	(chronic adj obstructive adj pulmonary adj disease).tw.
84	(respiratory adj disease *).tw.
85	exp Kidney Diseases/
86	(Kidney adj disease *).tw.
87	41 or 42 or 43 or 44 or 45 or 46 or 47 or 48 or 49 or 50 or 51 or 52 or 53 or 54 or 55 or 56 or 57 or 58 or 59 or 60 or 61 or 62 or 63 or 64 or 65 or 66 or 67 or 68 or 69 or 70 or 71 or 72 or 73 or 74 or 75 or 76 or 77 or 78 or 79 or80 or 81 or 82 or 83 or 84 or 85 or 86
88	40 and 87
89	Randomized Controlled Trials as Topic/
90	(Randomized adj controlled adj trial *).tw.
91	(Randomised adj controlled adj trial *).tw.
92	(Clinical adj Trial *).tw.
93	Random adj allocat *
94	(Clinical adj trial).pt.
95	(Controlled adj trial *).tw.
96	89 or 90 or 91 or 92 or 93 or 94 or 95
97	88 and 96
98	limit 97 to (English language and humans)

* represents wildcard symbol that broadens a search by finding words that start with the same letters.

**Table A2:** List of excluded articles from full-text screening stage with an overarching reason.

Articles	Number of Articles	Overarching Reason for Exclusion
(Aguiar, 2016; Bartels, 2004; Battersby, 2013; Bekelman, 2015; Berry, 2016; Brunisholz, 2017; Casas, 2006; de Stampa, 2014; Druss, 2001; Fors, 2015; Gjerdingen, 2009; Grochtdreis, 2018; Gums, 2016; Gums, 2014; Jakobsen, 2017; Jiao, 2014; Joubert, 2008; Kane, 2016; King, 2019; Ku, 2015; Peikes, 2009; Pourat, 2019; Schillinger, 2009; Siaw, 2018; Speyer, 2016; Walker, 2014; Wolff, 2010; Yoon, 2016; Yuting, 2017; Zatzick, 2015)	30	Participants: Patients less than 18 years; patients recruited and treated in a non-primary care setting; patients diagnosed with a communicable disease.
(Adam, 2010; Anderson, 2009; Borgermans, 2009; Campbell-Sills, 2016; Counsell, 2007; Eggers, 2018; Grunfeld, 2013; Ishani, 2016; Liu, 2003; Oosterbaan, 2013; Raftery, 1996; Rinfret, 2009; Rothman, 2005; Tao, 2015; Uittenbroek, 2017; Vermunt, 2012)	16	Intervention: Does not meet the PCMH definition or not sufficient components of PCMH or more focus on other intervention than PCMH model.
(Anjara, 2019; Bauer, 2019; Callahan, 2006; Ell, 2010; Hedrick, 2003; Jaen, 2010; Kearns, 2017; Kuhmmer, 2016; Meredith, 2016; Meulepas, 2007; Moran, 2011)	11	Comparison: Does not have a comparison group or comparison group received some amount of intervention other than standard care.
(Dwight-Johnson, 2010; Gill, 2017; Griffiths, 2016; Harpole, 2005; Marsteller, 2010; Marsteller, 2013)	6	Irrelevant outcomes
(Areán, 2005; Areán, 2007; Boland, 2015; Boult, 2013; Boyd, 2010; Buist-Bouwman, 2005; Campbell-Scherer, 2018; Chan, 2011; Conn, 2005; Ell, 2012; Ell, 2011; Fann, 2009; Ford, 2019; Fortney, 2014; Gensichen, 2006; Gilbody, 2007; Goering, 2003; Goertz, 2016; Hegel, 2005; Hendricks, 2016; Hirsch, 2014; Houles, 2010; Hunkeler, 2006; Jansen, 2017; Katon, 2006; Katon, 2003; Khambaty, 2015; Kinder, 2006; Kindy, 2003; Kumar, 2005; Lewis, 2017;Lin, 2014; McCusker, 2019; McGregor, 2011; Menchetti, 2013; Mills, 2003; Pieters, 2002; Price, 2004; Romano, 2011; Ruescas-Escolano, 2014; Sepers, 2015; Slimmer, 2003; Spoorenberg, 2016; Stone, 2010; Turner, 2011; Uittenbroek, 2017; Unutzer, 2001; Unutzer, 2006; Upchurch, 2005; Vester, 2019; Wang, 2011; Williams Jr, 2004; Zulman, 2015)	53	Other reasons: Non-English, conference abstracts, secondary data analyses using same sample, duplicate with different title, design and early implementation experiences paper, thesis, commentary, same outcome with same sample but different follow-up times.

**Table A3:** Characteristics of randomised controlled trials reviewed.

						Chronic Physical Conditions—Baseline Characteristics (Risk Proportion/Mean or Median and SD)				Outcomes				
Authors and Year of Publication	Country of Origin	Sample Size (N)	Mean Age/Age Groups	Gender Distribution (Female)	Chronic Disease Profile of the Sample Population	Treatment Group	Control Group	PCMH Components	Duration of Follow-up	Depression	Quality of Life/Self-Management	Hospital Admission	Cost/Health Utility	Biomedical Outcomes
Alexopoulos et al., 2009 [36]	United States	Treatment = 320 Control = 279	Overall ≥ 60 years (mean not reported)	Overall = 71.6%	Major or minor depression according to DSM-IV criteria	HAM-D score = 18.61 (6.12) Prevalence of suicide ideation = 27.5%	HAM-D score = 17.51 (5.82) Prevalence of suicide ideation = 18.6%	Team based care; Co-ordinated care	24 months	✓				
Aragonès et al., 2014 [18]	Spain	Treatment = 189 Control = 149	Overall = 47 years	Overall = 80%	Moderate or severe major depressive episode or minor depression	PHQ-9 score = 18.10 (5.20) SF12 mental health = 22.26 (9.05) SF12 physical health = 47.47 (10.98)	PHQ-9 score = 17.66 (4.79) SF12 mental health = 22.73 (10.44) SF12 physical health = 48.23 (11.23)	Team based care; Co-ordinated care; Patient engagement; Continuity of care.	36 months	✓	✓			
Aragonès et al., 2014 (Cost-effectiveness) [37]	Spain	Treatment = 189 Control = 149	Overall = 47 years	Overall = 80%	Moderate or severe major depressive episode or minor depression	Total direct costs—776.30 (664.10) Total indirect costs—718.30 (1587.70)	Total direct costs—593.80 (603.10) Total indirect costs—743.40 (1582.10)	Team based care; Co-ordinated care; Patient engagement; Continuity of care.	36 months				✓	
Aragonès et al., 2019 [38]	Spain	Treatment = 167 Control = 161	Treatment = 61.4 years Control = 59.3 years	Treatment = 82.6% Control = 83.2%	Major depressive episode and experiencing moderate or severe musculoskeletal pain.	HSCL-20 score; mean (SD) = 1.67 (0.80) BPI score; mean (SD) = 6.45 (1.87)	HSCL-20 score; mean (SD) = 1.69 (0.68) BPI score; mean (SD) = 6.60 (1.77)	MDT care, Patient engagement Coordinated care, Continuity of care	12 months	✓				
Barcelo et al., 2010 [39]	Mexico	Treatment = 196 Control = 111	6% of <40 years; 54% of 40–59 years; and 42% of ≥60 years	NA (baseline stratified by gender)	Type 2 Diabetes	% with HbA1c (<7%) Cases: Baseline—27.6%	% with HbA1c (<7%) Control: Baseline—20.7%	MDT care, All other components of CCM	13 months					✓
Bjorkelund et al., 2018 [40]	Sweden	Treatment = 192 Control = 184	Treatment = 40.8 years Control = 41.6 years	Treatment = 68.2% Control = 74.5%	Mild or moderate Depression	MADRS-S Mean (SD) = 20.8 (7.2) BDI-II Mean (SD) = 23.9 (8.7) EQ5D Mean (SD) = 0.58 (0.24)	MADRS-S Mean (SD) = 21.9 (7.1) BDI-II Mean (SD) = 25.1 (8.5) EQ5D Mean (SD) = 0.56 (0.25)	MDT care, Patient engagement Coordinated care	6 months	✓	✓			
Blom et al., 2016 [41]	Netherlands	Treatment = 3145 Control = 4133	Treatment = 80.5 years Control = 81.3 years	Treatment = 60.9% Control = 61.7%	Depression with complex daily functioning problems	Cantri’s ladder median (range) = 7 (6–8) GARS total score median (range) = 36 (27,45) BADL subscale score median (range) = 11 (9,15) IADL subscale score median (range) = 18 (25,30)	Cantri’s ladder median (range) = 7 (6–8) GARS total score median (range) = 37 (29,46) BADL subscale score median (range) = 11 (9,15) IADL subscale score median (range) = 20 (26,32)	MDT care, Self-management plans, Coordinated care	12 months		✓			
Bogner et al., 2008 [42]	United States	Treatment = 32 Control = 32	Treatment = 59.7 years Control = 57.5 years	Treatment = 75% Control = 78.1%	Depression and hypertension	CES-D mean score (SD) = 17.5 (13.2) SBP, mean (SD) = 146.7 (20.9) DBP, mean (SD) = 83.0 (10.7)	CES-D mean score (SD) = 19.6 (14.2) SBP, mean (SD) = 143.1 (22.5) DBP, mean (SD) = 81.4 (11.1)	MDT care, Patient engagement	6 weeks	✓				✓
Bogner et al., 2012 [43]	United States	Treatment = 92 Control = 88	Treatment = 57.8 years Control = 57.1 years	Treatment = 70% Control = 66%	Type 2 Diabetes, current prescription for antidepressant.	HbA1c, mean (SD) = 7.2 (1.8) PHQ-9 score, mean (SD) = 10.6 (7.9)	HbA1c, mean (SD) = 7.0 (1.9) PHQ-9 score, mean (SD) = 9.9 (7.2)	MDT care, Patient engagement	12 weeks	✓				✓
Boland et al., 2015 [44]	Netherlands	Treatment = 554 Control = 532	Treatment = 68.2 years Control = 68.4 years	Treatment = 49.5% Control = 42.7%	Chronic obstructive pulmonary disease according to GOLD (Global Initiative for COPD) guidelines.	CCQ score, mean (SD) = 1.54 (0.98)	CCQ score, mean (SD) = 1.46 (0.96)	MDT care, Self-management plans, Coordinated care	24 months				✓	
Borenstein et al., 2003 [45]	United States	Treatment =98 Control = 99	Treatment = 62.5 years Control = 61.5 years	Treatment = 63.2% Control = 58.5%	Hypertension	Mean SBP = 162 Mean DBP = 92 (no SD or 95% CI reported)	Mean SBP = 156 Mean DBP = 90 (no SD or 95% CI reported)	MDT care Patient education	12 months					✓
Bosanquet et al. 2017 [46]	United Kingdom	Treatment = 198 Control = 217	Treatment = 72 years Control = 72 years	Treatment = 59% Control = 63%	Depression	PHQ-9 score Mean (SD) = 12.3 (5.43)	PHQ-9 score Mean (SD) = 12.0 (5.32)	MDT care, Self-management plans, Coordinated care	18 months	✓	✓		✓	
Boult et al., 2008 [47]	United States	Treatment = 485 Control = 419	Treatment = 77.2 years Control = 78.1 years	Treatment = 54.2% Control = 55.4%	Multimorbidity (specific conditions not reported)	PACIC aggregate score = 5.9	PACIC aggregate score = 2.9	MDT care, Self-management plans, Coordinated care	6 months		✓			
Boult et al., 2011 [48]	United States	Treatment = 446 Control = 404	Treatment = 77.1 years Control = 77.8 years	Treatment = 54.3% Control = 55.7%	Circulatory system disorders, musculoskeletal disorders, Type 2 Diabetes, and cancers	No. of chronic diseases, mean (range) = 4.3 (1–11)	No. of chronic diseases, mean (range) = 4.3 (0–12)	MDT care, Self-management plans, Coordinated care	6 months			✓		
Callahan et al., 2005 [49]	United States	Treatment = 906 Control = 895	Treatment = 71 years Control = 71.4 years	Treatment = 64.1% Control = 65.6%	Major depression and/or dysthymia	SF-12 Mean (SD) = 40.43 (7.44) IADL Mean (SD) = 0.68 (1.37)	SF-12 Mean (SD) = 40.11 (7.40) IADL Mean (SD) = 0.61 (1.31)	MDT care, Patient engagement Coordinated care	12 months		✓			
Camacho et al., 2018 [13]	United Kingdom	Treatment = 191 Control = 196	Treatment = 57.9 years Control = 59.2 years	Treatment = 41% Control = 35%	Diabetes and/or coronary heart disease	SCL-D13 Mean (SD) = 2.364 (0.696)	SCL-D13 Mean (SD) = 2.330 (0.822)	MDT care, Patient engagement Coordinated care	24 months	✓			✓	
Campins et al., 2017 [20]	Spain	Treatment = 252 Control = 251	Treatment = 79.2 years Control = 78.8 years	Treatment = 60.3% Control = 57.4%	Patients with multimorbidity and polymedicated	Medications Mean (SD) = 10.79 (2.52)	Medications Mean (SD) = 10.91 (2.65)	MDT care, Patient engagement Coordinated care	12 months			✓	✓	
Chaney et al., 2011 [50]	United States	Treatment = 288 Control = 258	Treatment = 64 years Control = 64.4 years	Treatment = 4.2% Control = 3.5%	Subthreshold depression or dysthmia	PHQ-9 score Mean (SD) = 15.5 (4.4) SF-12 role physical score Mean (SD) = 29.2 (36.2) SF-12 role emotional score Mean (SD) = 47.1 (41.4)	PHQ-9 score Mean (SD) = 15.7 (4.7) SF-12 role physical score Mean (SD) = 34.8 (40.7) SF-12 role emotional score Mean (SD) = 50.0 (41.8)	MDT care, Patient engagement Coordinated care	7 months	✓	✓			
Cooper et al., 2013 [51]	United States	Treatment = 67 Control = 65	Treatment = 45.9 years Control = 47 years	Treatment = 55% Control = 50%	Major depressive disorder	CESD score, mean (SD) = 29.52 (14.48) MCS-12 score, mean (SD) = 35.97 (13.10)	CESD score, mean (SD) = 30.17 (13.78) MCS-12 score, mean (SD) = 36.41 (12.19)	MDT care, Patient engagement Coordinated care	12 months	✓	✓			
Coventry et al., 2015 [14]	United Kingdom	Treatment = 191 Control = 196	Treatment = 57.9 years Control = 59.2 years	Treatment = 41% Control = 35%	Diabetes and/or coronary heart disease	SCL-D-13 Mean (SD) = 2.36 (0.70) PHQ-9 Mean (SD) = 16.4 (4.2) GAD-7 Mean (SD) = 12.3 (5.1)	SCL-D-13 Mean (SD) = 2.33 (0.82) PHQ-9 Mean (SD) = 16.5 (4.1) GAD-7 Mean (SD) = 11.9 (5.3)	MDT care, Patient engagement Coordinated care	4 months	✓				
Dickinson et al., 2010 [52]	United States	Treatment = 187 Control = 214	Treatment = 62.1 years Control = 61.3 years	Treatment = 8% Control = 8%	Musculoskeletal disorders with chronic pain	RMDQ Mean (SD) = 14.9 (4.4) Pain disability-free days 0–3 months = 31.3 (25.3)	RMDQ Mean (SD) = 14.5 (4.4) Pain disability-free days 0–3 months = 30.0 (26.6)	MDT care, Patient engagement Coordinated care	12 months				✓	✓
Dobscha et al., 2009 [53]	United States	Treatment = 187Control = 214	Treatment = 62.1 years Control = 61.3 years	Treatment = 8% Control = 8%	Musculoskeletal disorders with chronic pain	RMDQ Mean (SD) = 14.9 (4.4) Current pain intensity, mean (SD) = 5.3 (2.2) PHQ-9 score Mean (SD) = 8.1 (5.7)	RMDQ Mean (SD) = 14.5 (4.4) Current pain intensity, mean (SD) = 5.1 (2.1) PHQ-9 score Mean (SD) = 8.4 (6.0)	MDT care, Patient engagement Coordinated care	12 months	✓	✓			✓
Dorr et al., 2008 [54]	United States	Treatment = 1144 Control = 2288	Treatment = 76.2 years Control = 76.2 years	Treatment = 64.6% Control = 64.6%	Circulatory system disorders, depression, and Type 2 Diabetes	Hospitalizations Mean (SD) = 257 (22.5) ED visits in previous year Mean (SD) = 407 (35.5)	Hospitalizations Mean (SD) = 514 (22.5) ED visits in previous year = 807 (35.3)	MDT care, Patient engagement Coordinated care, Data driven quality of care	24 months			✓		
Edelman et al., 2010 [16]	United States	Treatment = 133 Control = 106	Treatment = 63 years Control = 60.8 years	Treatment = 4.5% Control = 3.8%	Diabetes and hypertension	HbA1c % Mean (SD) = 9.2 (1.3) Mean SBP (SD) mmHg = 153.7 (14.8) Mean DBP (SD) mmHg = 84.7 (12.1)	HbA1c % Mean (SD) = 9.2 (1.5) Mean SBP (SD) mmHg = 153.7 (14.8) Mean DBP (SD) mmHg = 84.7 (12.1)	MDT care, Patient engagement Coordinated care	12 months					✓
Engel et al., 2016 [55]	United States	Treatment = 332 Control = 334	Treatment = 30.9 years Control = 31.4 years	Treatment = 80% Control = 82%	Posttraumatic Stress Disorder and Depression	PTSD severity, mean (SD) = 29.4 (9.4) SCL-20, mean (SD) = 2.1 (0.6)	PTSD severity, mean (SD) = 28.9 (8.9) SCL-20, mean (SD) = 2.0 (0.7)	MDT care, Patient engagement Coordinated care, Data driven quality of care	12 months	✓				
Fihn et al., 2011 [56]	United States	Treatment = 344 Control = 359	Treatment = 68.3 years Control = 67.2 years	Treatment = 1.2% Control = 3.6%	Circulatory system disorders—Angina	SAQ anginal frequency score, mean (SD) = 52.8 (17.3)	SAQ anginal frequency score, mean (SD) = 53.8 (16.5)	MDT care, Patient engagement Coordinated care	12 months					✓
Gilbody et al., 2017 [57]	United Kingdom	Treatment = 274 Control = 327	Treatment = 76.6 years Control = 77.4 years	Treatment = 55.5% Control = 62.4%	Subthreshold depression or dysthmia	PHQ-9 score, mean (SD) = 7.6 (4.32) Mean (SD) SF-12 score (physical component) = 38.5 (13.15)	PHQ-9 score, mean (SD) = 7.6 (4.55) Mean (SD) SF-12 score (physical component) = 36.6 (13.11)	MDT care, Self-management plans, Coordinated care	12 months	✓	✓			
Goorden et al., 2015 [58]	Netherlands	Treatment = 45 Control = 48	Treatment = 52 years Control = 53 years	Treatment = 66.7% Control = 72.9%	Major depressive disorder	Mean (SD) utility score EQ5D = 0.54 (0.25)	Mean (SD) utility score EQ5D = 0.56 (0.25)	MDT care, Patient engagement Coordinated care, Data driven quality of care	12 months		✓		✓	
Green et al., 2014 [59]	United Kingdom	Treatment = 276 Control = 305	Overall = 44.8 years	Overall = 71.9%	Depressive episode according to ICD-10	Mean (SD) utility score EQ5D = 0.504 (0.288)	Mean (SD) utility score EQ5D = 0.464 (0.313)	MDT care, Self-management plans, Coordinated care	12 months		✓		✓	
Grochtdreis et al., 2019 [60]	Germany	Treatment = 139 Control = 107	Treatment = 71.1 years Control = 71.6 years	Treatment = 77% Control = 79.4%	Depressive episode, recurring depressive disorder, or dysthmia according to ICD-10	EQ-5D-Index: mean (SD) = 0.55 (0.31) PHQ-9-Index: mean (SD) = 10.67 (4.02) Total costs: Mean (SD) = €2920 (€4425)	EQ-5D-Index: mean (SD) = 0.55 (0.31) PHQ-9-Index: mean (SD) = 9.64 (3.62) Total costs: Mean (SD) = €4222 (€7729)	MDT care, Patient engagement Coordinated care, Continuity of care	12 months		✓		✓	
Hirsch et al., 2014 [61]	United States	Treatment = 75 Control = 91	Treatment = 65.4 years Control = 69.6 years	Treatment = 60% Control =71 %	Diabetes and hypertension	Systolic BP (mmHg)—mean (SD) = 134.8 (17.4) Diastolic BP (mmHg)—mean (SD) = 75.1 (12.5)	Systolic BP (mmHg)—mean (SD) = 134.4 (16.5) Diastolic BP (mmHg)—mean (SD) = 75.7 (13.4)	MDT care, Patient engagement Coordinated care	9 months					✓
Hsu et al., 2014 [62]	Taiwan	Treatment = 789 Control = 271	NA	NA	Type 2 Diabetes	Mean (SD) HbA1c % = 8.4	Mean (SD) HbA1c % = 8.6	MDT care, Patient engagement Coordinated care	42 months					✓
Huijbregts et al., 2013 [63]	Netherlands	Treatment = 101 Control = 49	Treatment = 47 years Control = 52.1 years	Treatment = 72.3% Control = 73.5%	Major depressive disorder	Mean (SD) PHQ-9 = 15.5 (4.8)	Mean (SD) PHQ-9 = 14.8 (4.8)	MDT care, Patient engagement Coordinated care, Data driven quality of care	12 months	✓				
Ip et al., 2013 [64]	United States	Treatment = 147 Control = 147	Treatment = 55.5years Control = 57.2 years	Treatment = 12% Control = 12%	Type 2 Diabetes	Mean (SD) HbA1c % = 9.5 (1.4) Mean SBP (SD) mmHg = 128.9 (16.2) Mean DBP (SD) mmHg = 73.9 (9.8)	Mean (SD) HbA1c % = 9.3 (1.5) Mean SBP (SD) mmHg = 131 (14.8) Mean DBP (SD) mmHg = 76.6 (11.6)	MDT care, Patient engagement Coordinated care	12 months					✓
Johnson et al., 2016 [65]	United States	Treatment = 95 Control = 71	Treatment = 57 years Control = 63.4 years	Treatment = 58% Control = 40%	Type 2 Diabetes with depressive symptoms	PHQ, mean (SD) = 14.5 (3.8)	PHQ, mean (SD) = 14.2 (3.4)	MDT care, Patient engagement Coordinated care	12 months				✓	
Katon et al., 1999 [67]	United States	Treatment = 114 Control = 114	Treatment = 47.2 years Control = 46.7 years	Treatment = 67.5% Control = 81.6%	Depression or anxiety	SCL-depression mean (SD) = 1.9 (0.5)	SCL-depression mean (SD) = 1.9 (0.5)	MDT care, Patient engagement Coordinated care	6 months	✓				
Katon et al., 2004 [70]	United States	Treatment = 164 Control = 165	Treatment = 58.6 years Control = 58.1 years	Treatment = 65.2% Control = 64.8%	Diabetes and depression	SCL-20 score, mean (SD) = 1.7 (0.51)	SCL-20 score, mean (SD) = 1.6 (0.45)	MDT care, Patient engagement Coordinated care	12 months	✓				
Katon et al., 2005 [69]	United States	Treatment = 906 Control = 895	Treatment = 71 years Control = 71.4 years	Treatment = 64% Control = 66%	Major depression and/or dysthymia	Mean (SE) SCL-20 Depression Scores = 1.7 (0.6)	Mean (SE) SCL-20 Depression Scores = 1.7 (0.6)	MDT care, Patient engagement Coordinated care	24 months				✓	
Katon et al., 2010 [68]	United States	Treatment = 106 Control = 108	Treatment = 57.4 years Control = 56.3 years	Treatment = 48% Control = 56%	Diabetes, coronary heart disease, depression, and hypertension	SCL-20 mean (SD) = 1.7 (0.6) Glycated haemoglobin % mean (SD)= 8.1 (2.0) LDL cholesterol mg/dl mean (SD)= 106.5 (35.3) Systolic blood pressure mm Hg mean (SD)= 136 (18.4)	SCL-20 mean (SD) = 1.7 (0.6) Glycated haemoglobin % mean (SD)= 8.0 (1.9) LDL cholesterol mg/dl mean (SD)= 109.0 (36.5) Systolic blood pressure mmHg mean (SD)= 132 (17.2)	MDT care, Patient engagement Coordinated care	12 months	✓	✓			✓
Katon et al., 2012 [66]	United States	Treatment = 106 Control = 108	Treatment = 57.4 years Control = 56.3 years	Treatment = 48% Control = 56%	Diabetes and/or coronary heart disease	SCL-20 mean (SD) = 1.7 (0.6) PHQ-9 mean (SD) = 14.7 (3.8) SBP mean (SD) = 136 (18.4) HbA1c mean (SD) = 8.1 (2.0) Outpatient costs in the previous 12 months, mean (95% CI), $ = 10,026 (8312–11,741) Inpatient costs in the previous 12 months, mean (95% CI), $ = 3210 (1553–4868)	SCL-20 mean (SD) = 1.7 (0.6) PHQ-9 mean (SD) = 13.9 (3.1) SBP mean (SD) = 132 (17.2) HbA1c mean (SD) = 8.0 (1.9) Outpatient costs in the previous 12 months, mean (95% CI), $ = 9663 (8070–11,254) Inpatient costs in the previous 12 months, mean (95% CI), $ = 2748 (1453–4043)	MDT care, Patient engagement Coordinated care, Continuity of care	24 months				✓	
Konnopka et al., 2016 [22]	Germany	Treatment = 170 Control = 130	Treatment = 50.8 years Control = 46.1 years	Treatment = 75% Control = 75%	Depression and mild somatic symptom severity	PHQ-15 score, mean (SD) = 12.6 (4.73) SF-36 PCS, mean (SD) = 43.2 (9.1) SF-36 MCS, Mean (SD) = 41.5 (10.2)	PHQ-15 score, mean (SD) = 12.7 (4.86) SF-36 PCS, mean (SD) = 42.0 (8.9) SF-36 MCS, Mean (SD) = 40.7 (11.4)	MDT care, Patient engagement Coordinated care	12 months		✓		✓	
Krein et al., 2004 [71]	United States	Treatment = 123 Control = 123	Treatment = 61 years Control = 61 years	Treatment = 2% Control = 5 %	Type 2 Diabetes	Haemoglobin A1C (%) = 9.3 (1.5) LDL cholesterol (mg/dL) = 123 (37) Systolic blood pressure (mm Hg) = 145 (21) Diastolic blood pressure (mm Hg) = 86 (12)	Haemoglobin A1C (%) = 11 (9) LDL cholesterol (mg/dL) = 9.2 (1.4) Systolic blood pressure (mm Hg) = 123 (38) Diastolic blood pressure (mm Hg) = 145 (20)	MDT care, Patient engagement Coordinated care	18 months					✓
Kruis et al., 2014 [72]	Netherlands	Treatment = 554 Control = 532	Treatment = 68.2 years Control = 68.4 years	Treatment = 50.5 % Control = 57.3%	COPD according to GOLD (Global Initiative for COPD) guidelines.	Mean (SD) CCQ score Total = 1.5 (1.0) Mean (SD) SF-36 PCS = 38 (10.9) Mean (SD) SF-36 MCS = 48.3 (10.5) Mean (SD) PACIC score Total = 2.3 (0.9)	Mean (SD) CCQ score Total = 1.5 (1.0) Mean (SD) SF-36 PCS = 38.6 (10.7) Mean (SD) SF-36 MCS = 48.9 (10.3) Mean (SD) PACIC score Total = 2.3 (0.9)	MDT care, Patient engagement Coordinated care	24 months		✓			
Leeuwen et al., 2015 [73]	Netherlands	Treatment = 3017 Control = 1354	Overall = 80.5 years	Overall = 66.5%	Multimorbidity (specific conditions not reported) with high frailty index	EQ5D, mean (SD) = 0.60 (0.28)	EQ5D, mean (SD) = 0.59 (0.29)	MDT care, Patient engagement Coordinated care	24 months				✓	
Lin et al., 2000 [76]	United States	Treatment = 114 Control = 114	Treatment = 47.2 years Control = 46.7 years	Treatment = 67.5 % Control = 81.6%	Depression	Sheehan Disability Scale = 5.4 (5.0–5.8) SF-36 social functioning = 49.4 (44.6–54.2) SF-36 Role limitation due to emotional problems = 26.4 (21.1–31.7)	Sheehan Disability Scale = 5.3 (4.9–5.7) SF-36 social functioning = 49.4 (44.6–54.2) SF-36 Role limitation due to emotional problems = 26.4 (21.1–31.7)	MDT care, Patient engagement Coordinated care, Continuity of care	6 months		✓			
Lin et al., 2006 [74]	United States	Treatment = 506 Control = 495	Overall = 72 years	Overall = 68.3%	Major depression and/or dysthymia	Mean (SD) arthritis pain severity = 6.1 (2.7) Mean (SD) activity interference = 5.0 (3.2) Mean (SD) HSCL score = 1.7 (0.6)	Mean (SD) arthritis pain severity = 6.1 (2.7) Mean (SD) activity interference = 5.0 (3.2) Mean (SD) HSCL score = 1.7 (0.6)	MDT care, Patient engagement Coordinated care	12 months	✓				
Lin et al., 2012 [75]	United States	Treatment = 90 Control = 91	Overall = 56.8 years	Overall = 52.4%	Diabetes and/or coronary heart disease	Mean medication adherence Oral hypoglycaemic drugs = 0.83 (0.19) Antihypertensive = 0.85 (0.18) Lipid lowering = 0.82 (0.21) Antidepressant = 0.79 (0.23)	Mean medication adherence Oral hypoglycaemic drugs = 0.83 (0.20) Antihypertensive = 0.86 (0.18) Lipid lowering = 0.85 (0.18) Antidepressant = 0.80 (0.19)	MDT care, Patient engagement Coordinated care, Continuity of care	12 months				✓	
Maislos et al., 2004 [77]	Israel	Treatment = 48 Control = 34	Treatment = 58 years Control = 63 years	Treatment = 50 % Control = 65%	Type 2 Diabetes	Mean (SD) HbA1C, % = 11.6 (1.3)	Mean (SD) HbA1C, % = 11.1 (1.1)	MDT care, Patient engagement Coordinated care	6 months					✓
Menchetti et al., 2013 [78]	Italy	Treatment = 128 Control = 99	Treatment = 50.1 years Control = 53.9 years	Treatment = 78.9% Control = 72.7%	Depression	PHQ-9, Mean (SD) = 13.7 (4.7)	PHQ-9, Mean (SD) = 12.8 (4.6)	MDT care, Patient engagement Coordinated care	3 months	✓				
Metzelthin et al., 2015 [79]	Netherlands	Treatment = 103 Control = 91	Treatment = 77.5 years Control = 76.8 years	Treatment = 55% Control = 60%	Multimorbidity (specific conditions not reported) with high frailty index	GARS 18–72 = 33.1 (11.5) Mean EQ5D (SD) = 0.6 (0.2)	GARS 18–72 = 30.6 (10.6) Mean EQ5D (SD) = 0.7 (0.2)	MDT care, Patient engagement Coordinated care	24 months				✓	
Morgan et al., 2015 [80]	United States	Treatment = 269 Control = 165	Treatment = 79.1 years Control = 80.3 years	NA	Dementia	Charlson-Deyo index score Mean (SD) = 2.6 (2.4)	Charlson-Deyo index score Mean (SD) = 1.8 (1.7)	MDT care, Patient engagement Coordinated care	30 months				✓	
Muntingh et al., 2013 [19]	Netherlands	Treatment = 114 Control = 66	Treatment = 45 years Control = 49 years	Treatment = 73% Control = 61%	Panic and/or general anxiety disorders	Anxiety score (BAI) mean (SD) = 24.59 (11.52) Depression score (PHQ-9) mean (SD) = 9.40 (5.62) MCS (SF-36) mean (SD) = 32.56 (11.26) PCS (SF-36) mean (SD) = 48.43 (8.73) EQ-5D score mean (SD) = 0.67 (0.17)	Anxiety score (BAI) mean (SD) = 20.04 (11.28) Depression score (PHQ-9) mean (SD) = 8.98 (5.77) MCS (SF-36) mean (SD) = 35.74 (13.00) PCS (SF-36) mean (SD) = 47.75 (10.38) EQ-5D score mean (SD) = 0.70 (0.14)	MDT care, Patient engagement Coordinated care	12 months	✓	✓			
Pyne et al., 2003 [81]	United States	Treatment = 115 Control = 96	Treatment = 40 years Control = 47 years	Treatment = 83.5% Control = 85.4%	Major depressive disorder	Mean mCES-D (SD) = 57.6 (18.5) Mean VAS SF-36 (SD) = 0.453 (0.127)	Mean mCES-D (SD) = 50.8* (19.2) Mean VAS SF-36 (SD) = 0.446 (0.160)	MDT care, Patient engagement Coordinated care	12 months				✓	
Ramli et al., 2016 [82]	Malaysia	Treatment = 471 Control = 417	Treatment = 58 years Control = 57 years	Treatment = 62% Control = 64%	Type 2 Diabetes	HbA1c (%) = 8.4 (0.09) % HbA1c (≤7%) = 15.3	HbA1c (%) = 8.4 (0.09) % HbA1c (≤7%) = 17.0	MDT care, Patient engagement Coordinated care, Data driven quality of care	12 months					✓
Richards et al., 2008 [84]	United Kingdom	Treatment = 41 Control = 38	Treatment = 43 years Control = 43 years	Treatment = 78% Control = 76%	Depression	Mean (SD) PHQ-9 = 17.5 (4.9)	Mean (SD) PHQ-9 = 16.3 (4.5)	MDT care, Self-management plans, Coordinated care; Continuity of care	3 months	✓				
Richards et al., 2013 [83]	United Kingdom	Treatment = 276 Control = 305	Treatment = 45 years Control = 44.5 years	Treatment = 73.2% Control = 70.8%	Depression according to ICD-10	Mean (SD) PHQ-9 = 17.4 (5.2) Mean (SD) GAD- 7 = 12.9 (5.3) Mean (SD) SF-36 MCS = 23.2 (10.4) Mean (SD) SF-36 PCS = 44.8 (12.4)	Mean (SD) PHQ-9 = 18.1 (5.0) Mean (SD) GAD- 7 = 13.6 (4.7) Mean (SD) SF-36 MCS = 22.3 (10.3) Mean (SD) SF-36 PCS = 44.5 (12.3)	MDT care, Self-management plans, Coordinated care	12 months	✓	✓			
Rollman et al., 2005 [86]	United States	Treatment = 116 Control = 75	Treatment = 44 years Control = 45 years	Treatment = 84% Control = 77%	Panic and/or general anxiety disorders	Mean SIGH-A (SD) = 20.1 (6.4) Mean PDSS (SD) = 8.4 (6.0) Mean SF-12 MCS (SD) = 30.6 (8.8) Mean SF-12 PCS (SD) = 43.8 (11.8)	Mean SIGH-A (SD) = 20.6 (6.4) Mean PDSS (SD) = 8.5 (6.1) Mean SF-12 MCS (SD) = 29.9 (10.5) Mean SF-12 PCS (SD) = 45.1 (12.1)	MDT care, Self-management plans, Coordinated care	12 months	✓	✓			
Rollman et al., 2017 [85]	United States	Treatment = 124 Control = 126	Treatment = 45 years Control = 44.2 years	Treatment = 67% Control = 68%	Panic and/or general anxiety disorders	SF-36 MCS, mean (SD) = 27.4 (10.5) SF-36 PCS, mean (SD) = 45.6 (12.1) SIGH-A, mean (SD) = 28.4 (7.3) PDSS, mean (SD) = 12.8 (6.8) GADSS, mean (SD) = 15.9 (3.1) PHQ-9, mean (SD) = 15.2 (5.1)	SF-36 MCS, mean (SD) = 28.7 (9.9) SF-36 PCS, mean (SD) = 45.3 (11.7) SIGH-A, mean (SD) = 28.1 (6.5) PDSS, mean (SD) = 12.4 (6.4) GADSS, mean (SD) = 15.7 (3.2) PHQ-9, mean (SD) = 15.0 (5.1)	MDT care, Self-management plans, Coordinated care	24 months	✓				
Rollman et al., 2018 [87]	United States	Treatment = 302 Control = 101	Treatment = 43 years Control = 42 years	Treatment = 81% Control = 81%	Panic and/or general anxiety disorders	SF-12 MCS, mean (SD) = 31.7 (9.4) PROMIS Depression T-score, mean (SD) = 62.0 (6.3) PHQ-9 score, mean (SD) = 13.4 (4.7)	SF-12 MCS, mean (SD) = 31.1 (9.3) PROMIS Depression T-score, mean (SD) = 61.4 (6.4) PHQ-9 score, mean (SD) = 13.1 (4.9)	MDT care, Self-management plans, Coordinated care	6 months	✓				
Rost et al., 2001 [88]	United States	Treatment = 209 Control = 223	Overall = 43 years	Overall = 83.9%	Major depressive disorder	Mean mCESD = 56.9	Mean mCESD = 57.4	MDT care, Coordinated care	6 months	✓				
Salisbury et al., 2018 [89]	United Kingdom	Treatment = 797 Control = 749	Treatment = 71 years Control = 70.7 years	Treatment = 51% Control = 50%	At least three types of chronic condition—Circulatory system disorders, musculoskeletal disorders, Type 2 Diabetes, cancers, and mental illnesses	Mean (SD) EQ-5D-5L score = 0.574 (0.282) Mean (SD) PACIC score =	Mean (SD) EQ-5D-5L score = 0.542 (0.292) Mean (SD) PACIC score =	MDT care, Self-management plans, Coordinated care; Continuity of care	15 months		✓			
Scherpbier-de Haan et al., 2013 [90]	Netherlands	Treatment = 99 Control = 75	Treatment = 73.9 years Control = 72.4 years	Treatment = 62.2% Control = 47.3%	Depression and/or hypertension	Mean (SD) SBP = 142.7 (17.6) Mean (SD) DBP = 74.9 (9.2)	Mean (SD) SBP = 142.5(15.1 )Mean (SD) DBP = 80.4 (8.2)	MDT care, Self-management plans, Coordinated care	12 months					✓
Schnurr et al., 2013 [91]	United States	Treatment = 96 Control = 99	Treatment = 46.1 years Control = 44.4 years	Treatment = 7% Control = 10%	Posttraumatic Stress Disorder and Depression	PTSD Diagnostic Scale mean (SD)= 33.2 (8.3) Hopkins SCD mean (SD) = 1.98 (0.69) SF-36 Mental Component mean (SD) = 33.8 (8.8) SF-36 Physical Component mean (SD) = 42.2 (13.0)	PTSD Diagnostic Scale mean (SD)= 34.0 (9.7) Hopkins SCD mean (SD) = 2.06 (0.78) SF-36 Mental Component mean (SD) = 32.7 (8.1) SF-36 Physical Component mean (SD) = 43.4 (12.6)	MDT care, Self-management plans, Coordinated care	6 months	✓	✓			
Simon et al., 2001 [92]	United States	Treatment = 110 Control = 109	Overall = 47 years	Treatment = 67% Control = 82%	Depression	Mean number of depression-free days was 87.7 (95% CI = 76.6–96.7) for the collaborative care group	Mean number of depression-free days was 70.9 (95% CI = 60.8–81.3) for the usual care group	MDT care, Self-management plans, Coordinated care	6 months				✓	
Simon et al., 2004 [93]	United States	Treatment = 198 Control = 195	Treatment = 44.7 years Control = 44 years	Treatment = 74% Control = 78%	Depression	Mean (SD) SCL = 1.52 (0.58) Mean PHQ (SD) = 14.6 (5.1)	Mean (SD) SCL = 1.55 (0.62) Mean PHQ (SD) = 15.0 (5.5)	MDT care, Self-management plans, Coordinated care; Continuity of care	6 months	✓				
Simpson et al., 2011 [94]	Canada	Treatment = 131 Control = 129	Treatment = 58.8 years Control = 59.4 years	Treatment = 74% Control = 75%	Type 2 Diabetes	Mean (SD)SBP = 130.4 (14.9) Mean (SD) DBP = 74.4 (10.0)	Mean (SD) SBP = 128.3 (15.7) Mean (SD) DBP = 73.9 (10.8)	MDT care, Coordinated care	12 months					✓
Smith et al., 2004 [95]	Ireland	Treatment = 96 Control = 87	Treatment = 64.7 years Control = 65.6 years	Treatment = 54% Control = 57%	Type 2 Diabetes	Mean (SD) HbA1c (%) = 6.85% (1.6)	Mean (SD) HbA1c (%) = 6.6% (1.9)	MDT care, Coordinated care	12 months					✓
Tang et al., 2013 [96]	United States	Treatment = 202 Control = 213	Treatment = 54 years Control = 53.5 years	Treatment = 83% Control = 83%	Type 2 Diabetes	Mean (SD) HbA1c (%) = 9.28 (1.74)	Mean (SD) HbA1c (%) = 9.24 (1.59)	MDT care, Self-management plans, Coordinated care; Continuity of care; Data driven quality of care	12 months					✓
Taylor et al., 2005 [97]	Canada	Treatment = 20 Control = 19	Treatment = 58 years Control = 67 years	Treatment = 35% Control = 32%	Type 2 Diabetes	HbA1c (%) = 7.69 Systolic blood pressure (mm Hg) = 134 Diastolic blood pressure (mm Hg) = 79 Cholesterol (mg/dL) = 194.1 HDL cholesterol (mg/dL) = 44.9 LDL cholesterol (mg/dL) = 116 Triglycerides (mg/dL) = 205.5 (SD or 95% CI not reported)	HbA1c (%) = 7.69 Systolic blood pressure (mm Hg) = 129 Diastolic blood pressure (mm Hg) = 70 Cholesterol (mg/dL) = 201.01 HDL cholesterol (mg/dL) = 50.3 LDL cholesterol (mg/dL) = 119.1 Triglycerides (mg/dL) = 156.8 (SD or 95% CI not reported)	MDT care, Self-management plans, Coordinated care	4 months					✓
Thorn et al., 2020 [98]	United Kingdom	Treatment = 797 Control = 749	Treatment = 71 years Control = 70.7 years	Treatment = 51% Control = 50%	Three or more chronic conditions from those included in the National Health Service (NHS) Quality and Outcomes Framework—Circulatory system disorders, musculoskeletal disorders, Type 2 Diabetes, cancers, and mental illnesses	No. of long-term conditions from QOF: median (IQR) = 3.0 (3.0 to 3.0)	No. of long-term conditions from QOF: median (IQR) = 3.0 (3.0 to 3.0)	MDT care, Patient engagement Coordinated care, Continuity of care	6 months				✓	
Uijen et al., 2012 [99]	Netherlands	Treatment = 64 Control = 49	Treatment = 64 years Control = 63 years	Treatment = 58% Control = 75%	Chronic obstructive pulmonary disease according to ICD-10	Self-management group GOLD stage, n (%) GOLD 1 = 13 (20.3) GOLD 2 = 42 (65.6) GOLD 3/4 = 9 (14.1)	GOLD stage, n (%) GOLD 1 = 11 (22.4) GOLD 2 = 29 (59.2) GOLD 3/4 = 9 (18.4)	MDT care, Self-management plans, Coordinated care; Continuity of care;	24 months		✓			
Unutzer et al., 2002 [100]	United States	Treatment = 906 Control = 895	Treatment = 71.2 years Control = 71.4 years	Treatment = 64% Control = 66%	Major depression and/or dysthymia	Mean (SD) SCL-20 = 1.7 (0.6)	Mean (SD) SCL-20 = 1.7 (0.6)	MDT care, Patient engagement Coordinated care	12 months	✓	✓			
Unutzer et al., 2008 [101]	United States	Treatment = 279 Control = 272	Treatment = 72.6 years Control = 72.7 years	Treatment = 70% Control = 75%	Major depression and/or dysthymia	Depression severity score, mean (SD) = 1.7 (0.5)	Depression severity score, mean (SD) = 1.7 (0.6)	MDT care, Patient engagement Coordinated care	48 months				✓	
van Orden et al., 2009 [102]	Netherlands	Treatment = 102 Control = 63	Treatment = 40.2 years Control = 40.4 years	Treatment = 72% Control = 62%	Mental disorder	SCL-90 Mean (SD) = 181.2 (58.6) WHOQOL-BREF Mean (SD) = 3.0 (0.8)	SCL-90 Mean (SD) = 188.4 (64.2) WHOQOL-BREF Mean (SD) = 3.0 (1.0)	MDT care, Patient engagement Coordinated care	12 months	✓	✓			
Vera et al., 2010 [103]	Puerto Rico	Treatment = 89 Control = 90	Treatment = 57 years Control = 53 years	Treatment = 74% Control = 78%	Major depression and had any of the following health conditions: diabetes, hypothyroidism, asthma, hypertension, chronic bronchitis, arthritis, heart disease, high cholesterol, or stroke.	HSCL depression Mean (SD) = 2.22 (0.54)	HSCL depression Mean (SD) = 2.34 (0.58)	MDT care, Self-management plans, Coordinated care; Continuity of care;	6 months	✓				
Von Korff et al., 1998 [104]	United States	1st trial Treatment = 41 Control = 33 2nd trialTreatment = 26 Control = 31	NA	NA	Depression and on anti-depressant medications	Major depression Total depression treatment costs = $1337 Minor depression Total depression treatment costs = $1298	Major depression Total depression treatment costs = $850 Minor depression Total depression treatment costs = $656	MDT care, Patient engagement Coordinated care	12 months				✓	
Von Korff et al., 2011 [105]	United States	Treatment = 106 Control = 107	Treatment = 57.4 years Control = 56.3 years	Treatment = 48% Control = 56%	Diabetes, coronary heart disease, and depression	Sheehan social role disability scale = 5.6 (2.4) Global quality of life rating = 4.2 (1.9) WHODAS-2 activities of daily living = 15.8 (9.6)	Sheehan social role disability scale = 5.1 (2.6) Global quality of life rating = 4.7 (1.8) WHODAS-2 activities of daily living = 13.8 (9.6)	MDT care, Patient engagement Coordinated care, Continuity of care	12 months		✓			
Zwar et al., 2016 [106]	Australia	Treatment = 144 Control = 110	Treatment = 66.5 years Control = 65.4 years	Treatment = 61.1% Control = 58.2%	Chronic obstructive pulmonary disease	Mean total SGRQ score (SD) = 20.0 (17.2)	Mean total SGRQ score (SD) = 18.9 (16.8)	MDT care, Patient engagement Coordinated care	12 months		✓			✓

BADL—Basic Activities of Daily Living; BAI—Beck Anxiety Inventory; BP—blood pressure; CCM—chronic care model; CCQ—Clinical COPD questionnaire; CES-D—Center for Epidemiologic Studies Depression Scale; CI—confidence interval; DBP—diastolic blood pressure; DSM-IV—Diagnostic and Statistical Manual of Mental Disorders 4th edition; EQ3D—EuroQol 3 dimensions; EQ5D—EuroQol 5 dimensions; GAD—Generalized Anxiety Disorder; GADSS—Generalized Anxiety Disorder Severity Scale; GARS—Gilliam Autism Rating Scale; GOLD—Global initiative for Chronic Obstructive Lung Disease; HAM-D—Hamilton Depression Rating Scale; HbA1c—glycated haemoglobin; HDL—high density lipoprotein; HSCL—Hopkins Symptom Checklist; IADL—Instrumental Activities of Daily Living; ICD-10—10th revision of the International Statistical Classification of Diseases and Related Health Problems; IQR—interquartile range; LDL—low density lipoprotein; MADRS-S—Montgomery and Asberg Depression Rating Scale; MCS—mental component scores; MDT—multidisciplinary team; NA—not available; PACIC- Patient Assessment of Care for Chronic Conditions; PCS—physical component scores; PDSS—Panic Disorder Severity Scale; PHQ—Patient Health Questionnaire; PROMIS—Patient-Reported Outcomes Measurement Information System; PTSD—Post-traumatic stress disorder; QOF—Quality and Outcomes Framework; RMDQ—Roland-Morris Questionnaire; SAQ—Seattle Angina Questionnaire; SBP—systolic blood pressure; SD—standard deviation; SF 12 and SF 36—short and long format of a single measures of HRQoL.

**Table A4:** Characteristics of non-randomised controlled trials reviewed.

						Chronic Physical Conditions—Baseline Characteristics (Risk Proportion/Mean or Median and SD)				Outcomes				
Authors and Year of Publication	Country of origin	Sample Size (N)	Mean Age/Age Groups	Gender Distribution (Female)	Chronic Disease Profile of the Sample Population	Treatment Group	Control GROUP	PCMH Components	Duration of Follow-up	Depression	Quality of Life/Self-Management	Hospital Admission	Cost/Health Utility	Biomedical Outcomes
Bray et al., 2013 [17]	United States	Treatment = 368 Control = 359	Treatment = 59.5 years Control = 60.6 years	Treatment = 66% Control = 63%	Type 2 diabetes mellitus	HbA1c, mean (SD), % = 7.9 (2.2) SBP/DBP, mean (SD), mm Hg = 138 (18)/81 (10) HDL cholesterol, mean (SD), mg/dL= 50 (13.3) Total cholesterol, mean (SD), mg/dL = 176 (39.7)	HbA1c, mean (SD), % = 7.9 (2.2) SBP/DBP, mean (SD), mm Hg = 138 (18)/81 (10) HDL cholesterol, mean (SD), mg/dL= 50 (13.3) Total cholesterol, mean (SD), mg/dL = 176 (39.7)	6 key elements to the intervention design: education with behavioural coaching, treatment intensification, point-of-care management, expanded roles of clinic staff to facilitate management, a team care approach, and physician leadership	36 months					✓
Kravertz et al., 2016 [107]	United States	Treatment = 350 Control = 315	Treatment = 72.7 years Control = 72.2 years	NA	Hypertension	SBP = 167.7 DBP = 84 (SD or 95% CI not reported)	NA	MDT care, Patient education Coordinated care	4 months					✓
Petersen et al., 2019 [109]	South Africa	Treatment = 137 Control = 236	Treatment = 42.6 years Control = 44 years	Treatment = 83.2% Control = 80.5%	Mental and other comorbid conditions	PHQ-9 mean (SD) = 14.5 (3.47) WHODAS mean (SD) = 37.6 (17.19)	PHQ-9 mean (SD) = 12.8 (3.01) WHODAS mean (SD) = 40.0 (19.48)	MDT care, Patient engagement Coordinated care	12 months	✓				
Ruikes et al., 2016 [21]	Netherlands	Treatment = 287 Control = 249	Treatment = 83.1 years Control = 80.5 years	Treatment = 66.9% Control = 64.3%	Frail elderly people with multimorbidity	Katz-15 index, mean (SD) = 5.4 (2.9)	Katz-15 index, mean (SD) = 4.6 (2.7)	MDT care, Self-management plans, Coordinated care	12 months		✓	✓		
Seidu et al., 2017 [110]	United Kingdom	Treatment = 6054 Control = 2312	% above 65 years Treatment = 14.20 Control = 11.31	Treatment = 50.6% Control = 47.4%	Type 2 diabetes mellitus	Non-elective bed days, mean (SD) = 5.62 (2.11)	Non-elective bed days, mean (SD) = 3.82 (1.62)	MDT care, Self-management plans, Coordinated care	12 months			✓		
Sommers et al., 2000 [111]	United States	Treatment = 280 Control = 263	Treatment = 78 years Control = 77 years	1	Frail elderly people with multimorbidity	Hospital admissions per patient per year, mean (SD) = 0.34 (0.68) ≥1 hospital admission within 60 days % = 4.5 ≥1 ED visit % = 9.0	Hospital admissions per patient per year, mean (SD) = 0.39 (0.81) ≥1 hospital admission within 60 days % = 5.9 ≥1 ED visit % = 5.9	MDT care, Self-management plans, Coordinated care	24 months			✓		
Vestjens et al., 2019 [108]	Netherlands	Treatment = 232 Control = 232	Treatment = 82.4 years Control = 82.4 years	Treatment = 72.4% Control = 72.4%	Frail elderly people with multimorbidity	EQ5D3L = 0.63 (0.26)	EQ5D3L = 0.66 (0.24)	MDT care, Patient engagement Coordinated care	12 months		✓			

BP—blood pressure; CI—confidence interval; DBP—diastolic blood pressure; ED—emergency department; EQ3D—EuroQol 3 dimensions; HbA1c—glycated haemoglobin; HDL—high density lipoprotein; LDL—low density lipoprotein; MDT—multidisciplinary team; NA—not available; PHQ—Patient Health Questionnaire; SBP—systolic blood pressure; SD—standard deviation; WHODAS—World Health Organization Disability Assessment Schedule.

**Table A5:** Quality assessment of randomised controlled studies using Joanna Briggs Institute (JBI) critical appraisal checklist.

Author and Year	Q 1	Q 2	Q 3	Q 4	Q 5	Q 6	Q 7	Q 8	Q 9	Q 10	Q 11	Q 12	Q 13	Quality
Alexopoulos et al., 2009 [36]	U	U	N	NA	NA	U	Y	N	Y	Y	Y	Y	Y	Fair
Aragonès et al., 2014 [18]	U	U	Y	NA	NA	Y	Y	Y	Y	Y	Y	Y	Y	Good
Aragonès et al., 2019 [38]	Y	Y	Y	NA	NA	Y	Y	N	Y	Y	Y	Y	Y	Good
Barcelo et al., 2010 [39]	U	U	Y	NA	NA	U	Y	N	Y	Y	Y	U	Y	Fair
Bjorkelund et al., 2018 [40]	U	U	Y	NA	NA	U	Y	Y	Y	Y	Y	Y	Y	Good
Blom et al., 2016 [41]	U	U	Y	NA	NA	Y	Y	N	Y	Y	Y	Y	Y	Good
Bogner et al., 2008 [42]	U	U	Y	NA	NA	U	Y	N	Y	Y	Y	Y	Y	Fair
Bogner et al., 2012 [43]	Y	Y	Y	NA	NA	Y	Y	N	Y	Y	Y	Y	Y	Good
Borenstein et al., 2003 [45]	U	U	Y	NA	NA	U	Y	N	Y	Y	Y	U	Y	Fair
Bosanquet et al., 2017 [46]	Y	Y	Y	NA	NA	Y	Y	Y	Y	Y	Y	Y	Y	Good
Boult et al., 2008 [47]	U	U	Y	NA	NA	Y	Y	Y	Y	Y	Y	Y	Y	Good
Boult et al., 2011 [48]	U	U	Y	NA	NA	Y	Y	Y	Y	Y	Y	Y	Y	Good
Callahan et al., 2005 [49]	U	U	Y	NA	NA	U	Y	N	Y	Y	Y	Y	Y	Fair
Camacho et al., 2018 [13]	Y	Y	Y	NA	NA	Y	Y	N	Y	Y	Y	Y	Y	Good
Campins et al., 2017 [20]	Y	Y	Y	NA	NA	N	Y	N	Y	Y	Y	Y	Y	Good
Chaney et al., 2011 [50]	U	U	Y	NA	NA	U	Y	Y	Y	Y	Y	Y	Y	Good
Cooper et al., 2013 [51]	Y	Y	Y	NA	NA	N	Y	U	Y	Y	Y	Y	Y	Good
Coventry et al., 2015 [14]	Y	Y	Y	NA	NA	N	Y	Y	Y	Y	Y	Y	Y	Good
Dobscha et al., 2009 [53]	Y	Y	Y	NA	NA	Y	Y	Y	Y	Y	Y	Y	Y	Good
Dorr et al., 2008 [54]	U	U	Y	NA	NA	U	Y	N	U	Y	Y	Y	Y	Fair
Edelman et al., 2010 [16]	Y	Y	Y	NA	NA	Y	Y	U	U	Y	Y	Y	Y	Fair
Engel et al., 2016 [55]	Y	Y	Y	NA	NA	N	Y	N	Y	Y	Y	Y	Y	Good
Fihn et al., 2011 [56]	U	U	Y	NA	NA	N	Y	N	Y	Y	Y	Y	Y	Fair
Gilbody et al., 2017 [57]	Y	Y	Y	NA	NA	Y	Y	Y	Y	Y	Y	Y	Y	Good
Green et al., 2014 [59]	U	U	Y	NA	NA	N	Y	N	Y	Y	Y	Y	Y	Fair
Hirsch et al., 2014 [61]	Y	Y	Y	NA	NA	N	Y	N	Y	Y	Y	Y	Y	Good
Hsu et al., 2014 [62]	U	U	N	NA	NA	U	Y	N	U	Y	Y	Y	U	Poor
Huijbregts et al., 2013 [63]	Y	Y	Y	NA	NA	U	Y	Y	Y	Y	Y	Y	Y	Good
Ip et al., 2013 [64]	U	U	Y	NA	NA	U	Y	N	Y	Y	Y	Y	Y	Fair
Katon et al., 2012 [66]	U	U	Y	NA	NA	Y	Y	Y	Y	Y	Y	Y	Y	Good
Katon et al., 1999 [67]	Y	Y	Y	NA	NA	Y	Y	Y	Y	Y	Y	Y	Y	Good
Katon et al., 2010 [68]	Y	Y	Y	NA	NA	Y	Y	Y	Y	Y	Y	Y	Y	Good
Katon et al., 2004 [70]	Y	Y	Y	NA	NA	Y	Y	Y	Y	Y	Y	Y	Y	Good
Konnopka et al., 2016 [22]	U	U	Y	NA	NA	N	Y	N	Y	Y	Y	Y	Y	Fair
Krein et al., 2004 [71]	Y	Y	Y	NA	NA	Y	Y	Y	Y	Y	Y	Y	Y	Good
Kruis et al., 2014 [72]	Y	Y	Y	NA	NA	Y	Y	Y	Y	Y	Y	Y	Y	Good
Lin et al., 2000 [76]	Y	Y	Y	NA	NA	Y	Y	Y	Y	Y	U	Y	Y	Fair
Lin et al., 2006 [74]	U	U	U	NA	NA	U	Y	N	Y	Y	Y	Y	Y	Fair
Lin et al., 2012 [75]	Y	Y	Y	NA	NA	N	Y	N	Y	Y	Y	Y	Y	Good
Maislos et al., 2004 [77]	U	U	Y	NA	NA	U	Y	N	Y	Y	Y	Y	Y	Fair
Menchetti et al., 2013 [78]	Y	Y	Y	NA	NA	U	Y	Y	Y	Y	Y	Y	Y	Good
Muntingh et al., 2013 [19]	Y	Y	N	NA	NA	Y	Y	U	Y	Y	Y	Y	Y	Fair
Ramli et al., 2016 [82]	Y	Y	Y	NA	NA	U	Y	Y	Y	Y	Y	Y	Y	Good
Richards et al., 2013 [83]	Y	Y	Y	NA	NA	U	Y	Y	Y	Y	Y	Y	Y	Good
Richards et al., 2008 [84]	Y	Y	Y	NA	NA	Y	Y	Y	Y	Y	Y	Y	Y	Good
Rollman et al., 2005 [86]	Y	Y	Y	NA	NA	Y	Y	Y	Y	Y	Y	Y	Y	Good
Rollman et al., 2017 [85]	Y	Y	Y	NA	NA	Y	Y	Y	Y	Y	Y	Y	Y	Good
Rollman et al., 2018 [87]	Y	Y	Y	NA	NA	Y	Y	Y	Y	Y	Y	Y	Y	Good
Rost et al., 2001 [88]	N	N	Y	NA	NA	U	Y	N	Y	Y	Y	Y	Y	Fair
Salisbury et al., 2018 [89]	Y	Y	Y	NA	NA	Y	Y	Y	Y	Y	Y	Y	Y	Good
Scherpbier-de Haan et al., 2013 [90]	U	U	Y	NA	NA	N	Y	Y	Y	Y	Y	Y	Y	Good
Schnurr et al., 2013 [91]	Y	Y	Y	NA	NA	Y	Y	Y	Y	Y	Y	Y	Y	Good
Simon et al., 2004 [93]	Y	Y	Y	NA	NA	Y	Y	Y	Y	Y	Y	Y	Y	Good
Simpson et al., 2011 [94]	Y	Y	Y	NA	NA	Y	Y	Y	Y	Y	Y	Y	Y	Good
Smith et al., 2004 [95]	Y	Y	Y	NA	NA	U	Y	Y	Y	Y	Y	Y	Y	Good
Tang et al., 2013 [96]	Y	Y	Y	NA	NA	Y	Y	Y	Y	Y	Y	Y	Y	Good
Taylor et al., 2005 [97]	Y	Y	Y	NA	NA	N	Y	N	Y	Y	U	Y	Y	Good
Uijen et al., 2012 [99]	Y	Y	Y	NA	NA	Y	Y	Y	Y	Y	Y	Y	Y	Good
Unutzer et al., 2002 [100]	Y	Y	Y	NA	NA	Y	Y	Y	Y	Y	Y	Y	Y	Good
van Orden et al., 2009 [102]	Y	Y	Y	NA	NA	N	Y	N	Y	Y	Y	Y	Y	Good
Vera et al., 2010 [103]	Y	Y	Y	NA	NA	Y	Y	N	Y	Y	Y	Y	Y	Good
Von Korff et al., 2011 [105]	Y	Y	Y	NA	NA	N	Y	N	Y	Y	Y	Y	Y	Good
Zwar et al., 2016 [106]	Y	Y	Y	NA	NA	Y	Y	Y	Y	Y	Y	Y	Y	Good

NA—Most did not blind participants or personnel as it was not practical. Therefore, we did not downgrade for these risks/uncertainties.

**Table A6:** Quality assessment of non-randomised controlled studies using JBI critical appraisal checklist.

Author and Year	Q 1	Q 2	Q 3	Q 4	Q 5	Q 6	Q 7	Q 8	Q 9	Quality
Bray et al., 2013 [17]	Y	Y	Y	Y	Y	Y	Y	Y	Y	Good
Kravertz et al., 2016 [107]	Y	Y	Y	Y	Y	U	Y	U	U	Fair
Petersen et al., 2019 [109]	Y	Y	Y	Y	Y	Y	Y	Y	Y	Good
Ruikes et al., 2016 [21]	Y	Y	Y	Y	Y	Y	Y	Y	Y	Good
Seidu et al., 2017 [110]	Y	Y	Y	Y	Y	U	Y	U	U	Fair
Sommers et al., 2000 [111]	Y	Y	Y	Y	Y	U	Y	Y	Y	Good
Vestjens et al., 2019 [108]	Y	Y	Y	Y	Y	Y	Y	Y	Y	Good

**Table A7:** Quality assessment of studies on economic evaluation using JBI critical appraisal checklist.

Author and Year	Q 1	Q 2	Q 3	Q 4	Q 5	Q 6	Q 7	Q 8	Q 9	Q 10	Q 11	Quality
Aragonès et al., 2014 (Cost-effectiveness) [37]	Y	Y	Y	Y	Y	Y	Y	Y	Y	Y	Y	Good
Boland et al., 2015 [44]	Y	Y	Y	Y	Y	Y	Y	Y	Y	Y	N	Good
Dickinson et al., 2010 [52]	Y	Y	Y	Y	U	U	Y	Y	U	Y	U	Fair
Goorden et al., 2015 [58]	Y	Y	Y	Y	Y	Y	Y	Y	Y	Y	U	Good
Grochtdreis et al., 2019 [60]	Y	Y	Y	Y	Y	Y	Y	Y	Y	Y	N	Good
Johnson et al., 2016 [65]	Y	Y	Y	Y	Y	Y	Y	Y	Y	Y	Y	Good
Katon et al., 2005 [69]	Y	Y	Y	Y	Y	Y	Y	Y	Y	Y	U	Good
Leeuwen et al., 2015 [73]	Y	Y	Y	Y	Y	Y	Y	Y	Y	Y	Y	Good
Metzelthin et al., 2015 [79]	Y	Y	Y	Y	Y	Y	Y	Y	Y	Y	U	Good
Morgan et al., 2015 [80]	Y	Y	Y	Y	U	U	Y	N	N	Y	U	Fair
Pyne et al., 2003 [81]	Y	Y	Y	Y	Y	Y	Y	Y	Y	Y	U	Good
Simon et al., 2001 [92]	Y	Y	Y	Y	Y	Y	Y	Y	Y	Y	U	Good
Thorn et al., 2020 [98]	Y	Y	Y	Y	Y	Y	Y	Y	Y	Y	U	Good
Unutzer et al., 2008 [101]	Y	Y	Y	Y	U	U	Y	N	N	U	Y	Fair
Von Korff et al., 1998 [104]	Y	Y	Y	Y	U	U	U	N	N	Y	U	Poor

**Table A8:** Quality assessment of non-randomised controlled studies using Risk of Bias In Non-randomised Studies of Interventions (ROBINS-I) tool.

Author and Year	Q 1	Q 2	Q 3	Q 4	Q 5	Q 6	Q 7	Overall
Bray et al., 2013 [17]	Low	Low	Low	Low	Low	Low	Low	Good
Kravertz et al., 2016 [107]	Moderate	Low	Low	Low	Low	Low	Moderate	Fair
Petersen et al., 2019 [109]	Low	Low	Low	Low	Low	Low	Low	Good
Ruikes et al., 2016 [21]	Low	Low	Low	Low	Low	Low	Low	Good
Seidu et al., 2017 [110]	Moderate	Low	Low	Low	Low	Low	Moderate	Fair
Sommers et al., 2000 [111]	Low	Low	Low	Low	Low	Low	Low	Good
Vestjens et al., 2019 [108]	Low	Low	Low	Low	Low	Low	Low	Good
